# Comparative Analysis of Seventeen Mitochondrial Genomes of Mileewinae Leafhoppers, Including the Unique Species *Mileewa digitata* (Hemiptera: Cicadellidae: Mileewinae) From Xizang, China, and New Insights Into Phylogenetic Relationships Within Mileewini

**DOI:** 10.1002/ece3.70830

**Published:** 2025-01-09

**Authors:** Hongli He, Bin Yan, Xiaofei Yu, Maofa Yang

**Affiliations:** ^1^ Institute of Entomology of Guizhou University Guizhou Provincial Key Laboratory for Agricultural Pest Management of the Mountainous Region Guiyang Guizhou China; ^2^ College of Tobacco Sciences of Guizhou University Guiyang Guizhou China

**Keywords:** ancestral state, comparative analysis, Mileewinae, Mileewini, mitochondrial genome, phylogenetics

## Abstract

The subfamily Mileewinae in China comprises one tribe (Mileewini), four genera (*Ujna*, *Mileewa*, *Processina*, *Anzihelus*), and 71 species, yet only 11 mitochondrial genomes have been published. This study aimed to elucidate ambiguous diagnostic traits in traditional taxonomy and examined phylogenetic relationships among genera by sequencing mitochondrial genomes from 16 species. The lengths of the mitochondrial genomes ranged from 14,532 to 15,280 bp, exhibiting an AT content of 77.2%–80.7%, indicative of AT bias. Each genome contains 13 protein‐coding genes (PCGs), 22 transfer RNA genes (tRNAs), and 2 ribosomal RNA genes (rRNAs). Notably, the genome of the Xizang endemic *Mileewa digitata* measures 15,280 bp, with an AT content of 80.7%, 10,941 bp of PCGs, and a 949 bp control region (CR) followed by a 19 bp poly‐A tail. Gene arrangement among the 17 genomes mirrors that of other Mileewinae species. Analyses of nucleotide diversity and evolutionary rates indicate purifying selection on PCGs, with atp8 exhibiting the greatest variability and evolutionary rate, whereas cox1 shows the least. Genetic distance studies display minimal separation among *Mileewa* species, specifically *Mileewa nii* with *Mileewa margheritae* and *Mileewa rufivena* with *Mileewa ussurica*, as well as the two morphs of *Mileewa digitata*. Phylogenetic analyses using Bayesian Inference (BI) and Maximum Likelihood (ML) generated six trees, further questioning the monophyly of the genera *Mileewa*, *Ujna*, and *Processina*. The reconstructed ancestral state based on the forewing patch position suggests that the common ancestor of Chinese Mileewini species was situated exclusively on the costal margin, prompting a proposed division of the tribe's species into two categories (genera). This research enriches the understanding of phylogenetic relationships within Mileewinae and contributes to the mitochondrial genome database for this group, paving the way for future taxonomic studies in China.

## Graphical abstract

In this study, we sequenced and comparatively analyzed 17 mitochondrial genomes from 16 species of Mileewinae, including the distinct mitochondrial genome of the unique species *Mileewa digitata*. Phylogenetic analyses employing Bayesian Inference (BI) and Maximum Likelihood (ML) methods produced six trees that further challenge the monophyly of the genera *Mileewa*, *Ujna*, and *Processina*. The reconstructed ancestral state based on the forewing patch position suggests that the common ancestor of Chinese Mileewini species was situated exclusively on the costal margin, prompting a proposed division of the tribe's species into two categories (genera).

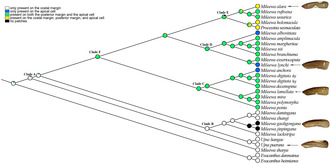

## 1 Introduction

Cicadellidae is the largest family with 25 subfamilies in Hemiptera, comprising over 22,000 species worldwide (Dietrich 2004, 2005). As one member of Cicadellidae, the small leafhopper subfamily Mileewinae just encompasses approximately 160 described species globally. In previous classification studies, there were some controversies, leading to the placement of subfamily Mileewinae species under both the subfamilies Cicadellinae and Typhlocybinae (Evans 1947; Young 1965, 1968; Mahmood 1967; Dietrich et al. 2005). It was not until Dietrich et al. proposed a widely accepted classification system in 2011 that Mileewinae was considered as a separate subfamily and now includes four tribes: Makilingiini (found in the Philippines and Thailand), Mileewini (distributed in the Old and New World), Tinteromini and Tungurahualini (both found in the New World) (Dietrich 2011; Krishnankutty and Dietrich 2011). Among them, all species of Mileewinae in China belong to the tribe Mileewini. In recent years, several new species of the tribe Mileewini from China have been described and published, bringing the current number of Mileewinae species in China to 71 (Yu and Zhang 2022; Yan et al. 2021; He et al. 2021; He, Yan, and Yang 2021; Yu, He, and Yang 2021). All 71 species are classified into four genera: *Mileewa* Distant (1908), *Ujna* Distant (1908), *Processina* Yang, Deitz, and Li (2005), and *Anzihelus* Yan et al. (2021). The majority of Chinese Mileewini species are found in the genus *Mileewa*, which includes 56 species, followed by *Ujna* with eight species, genera *Processina* and *Anzihelus* comprise six and one species, respectively, both of which are recorded exclusively in China (He et al. 2021). Some recent phylogenetic study has also confirmed the monophyly of Mileewinae as a subfamily; however, the relationships of the three genera (*Ujna*, *Mileewa*, and *Processina*) of Mileewinae in China are still not well resolved (Yu and Zhang 2021; He et al. 2022; Dietrich et al. 2022). These small to medium‐sized, slender, darkly pigmented species predominantly inhabit wet tropical forests, where they are commonly found on herbaceous vegetation in the understory. Given their specific habitat and host plant requirements of the species in this subfamily, our previous research primarily focused on utilizing the limited specimens for traditional taxonomic species descriptions. Regarding the study of mitochondrial genomes, a total of 11 mitochondrial genomes of Mileewini were reported and deposited in NCBI database (Yu and Zhang 2021; He et al. 2022; He and Yang 2020a, 2020b, 2021; He, Li, and Yang 2019). However, considering that there are currently 71 species of this subfamily in China, the available mitochondrial genomes are still very limited.

Besides, there are still several issues that need to be explored in the taxonomic research of Mileewinae in China. First, some male adults of Mileewinae species exhibit polymorphism, displaying notable differences in body color, markings, individual size, and genitalia among individuals, such as *Mileewa digitata* (Yu, He, and Yang 2021) in this study. Second, there are some ambiguities regarding the distinguishing characteristics between the genera *Mileewa* and *Ujna*. Additionally, it remains to be determined whether the feature of having a style with a straight apical portion, rounded apex, and dense setae at the apex should be used to differentiate the genus *Processina* from the other two genera (*Ujna* and *Mileewa*). For instance, *Processina nigroscens* (Yang and Meng 2010), which was previously classified under the genus *Mileewa*, has been placed in the genus *Processina* due to its rounded apex characteristic of the style (Yang, Meng, and Li 2017). Third, there are several species that exhibit similar taxonomic characteristics, such as *Mileewa jinpingana* (Yang, Meng, and Li 2017), and *Mileewa gaoligongana* (Yang, Meng, and Li 2010), as well as *Mileewa margheritae* (Distant 1908), and *Mileewa nii* (Yang, Meng, and Li 2017). Therefore, it is essential to supplement with more mitochondrial genome molecular data for comparative analysis to identify their differences. At the same time, utilizing these data to resolve and verify the phylogenetic relationships among genera and species within the subfamily Mileewinae is crucial.

**FIGURE 1:**
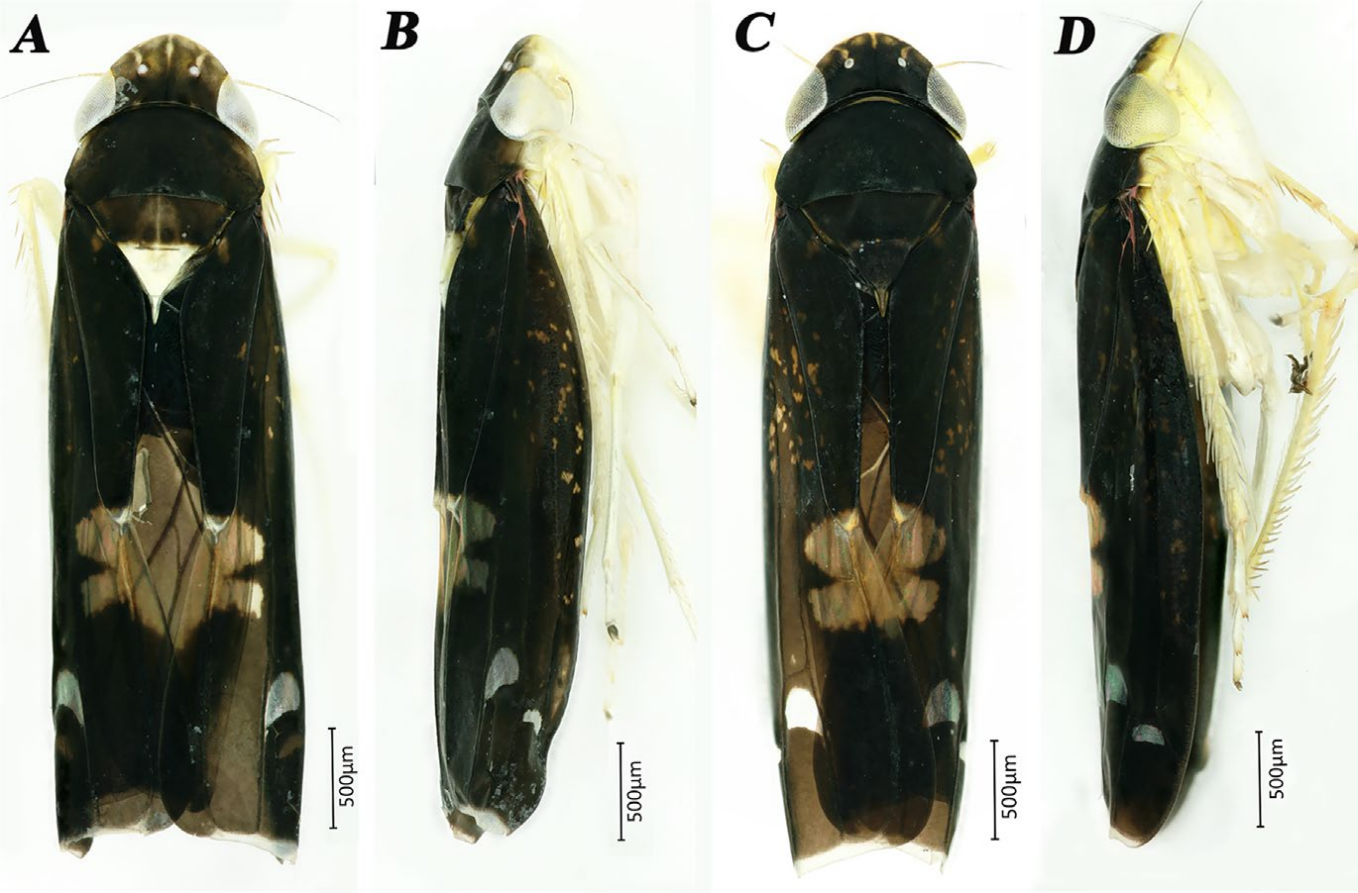
Male specimens exhibiting two distinct morphological forms of *Mileewa digitata*. A&B, *Mileewa digitata* dy. C&D *Mileewa digitata* hy. A&C Male adult, dorsal view. B&D Male adult, lateral view.

*Mileewa digitata*, which is an endemic species only distributed in Motuo county, Xizang, China (Yu, He, and Yang 2021). This species closely resembles *Mileewa octospina* (Yang, Meng, and Li 2010) in external morphological characteristics but differs from it in having a very slender ventral pygofer process and apical aedeagus processes that are extended laterally. The specimens of this species exhibit two distinct morphologies (represented by dy and hy), with their main differences outlined as follows: the head, thorax, and forewings are black. The upper half of the crown is brownish (black in hy), featuring three yellowish longitudinal stripes and several irregular cloudy spots. The distal half of the scutellum is yellow‐white, while the middle of the basal half is brownish‐yellow, marked by a distinct vertical line and two round black spots beside the end of the vertical line (showed in dy). In hy males, the thorax and scutellum are entirely black, lacking any stripes (Figure 1). Concurrently, variations in male genital traits exist between the two morphological individuals, including three distinct shapes of the aedeagus apex. One of the objectives of this study is to compare the mitochondrial genome differences between two distinct morphological forms of the species, to confirm the validity of the polymorphic phenomenon present in this species.

Due to their simple structure, short length, rapid evolutionary rate, and matrilineal inheritance, insect mitochondrial genomes are widely used as molecular markers in population genetic studies and phylogenetic analyses (Huang et al. 2022; Wolstenholme 1992; Zhang and Hewitt 1997). The insect mitochondrial genomes are mostly 14–18 kb double‐stranded circular molecules comprising 37 typical genes: 13 protein‐coding genes (PCGs), two ribosomal RNA genes (rrnL and rrnS), 22 transfer RNA genes (tRNAs), and a control region (CR) with variable length as the origin of transcription and replication (Wolstenholme 1992; Zhang and Hewitt 1997; Boore 1999; Cameron 2014). This study involved the sequencing and annotation of mitochondrial genomes for 16 species from the genera *Ujna* and *Mileewa* for the first time. However, owing to quality issues during sequencing, only one of the 17 genomes successfully yielded an assembled control region sequence. Furthermore, we conducted a comparative analysis of the characteristics of these mitogenomes, including nucleotide composition, tRNA secondary structure, codon usage, gene overlaps, intergenic spacers, and the CR of *Mileewa digitata* dy. Simultaneously, we employed mitochondrial genome data from 27 species, combining information from this study and prior research, to assess the ka/ks ratios, nucleotide diversity of the PCGs, and the genetic distances among the species. In the end, we analyzed the mitochondrial genome data from 27 Mileewinae species to reconstruct the phylogenetic relationships among both genera and species using multiple data sets. Additionally, we employed the resulting phylogenetic tree to infer the ancestral traits of the tribe Mileewini in China, focusing specifically on forewing morphology. The aim was to assist in the current taxonomic identification efforts and to provide new insights into the classification status of various genera.

## 2 Materials and Methods

### 2.1 Taxonomic Identification and DNA Extraction

The 17 male adult specimens of the genera *Mileewa* and *Ujna* are *Mileewa anchora*, *Mileewa branchiuma*, *Mileewa damingana*, *Mileewa digitata* dy, *Mileewa digitata* hy, *Mileewa exsertocaputa*, *Mileewa gaoligongana*, *Mileewa holomacula*, *Mileewa jinpingana*, *Mileewa lackstripa*, *Mileewa lynchi*, *Mileewa nii*, *Mileewa polymorpha*, *Mileewa decemspina*, *Mileewa ussurica*, *Mileewa zhangi*, *Ujna liangae*, both collected from China, with specific collection information provided in Table 1. All specimens were collected, immediately preserved in anhydrous ethanol, and stored at −20°C in a laboratory freezer prepared for DNA extraction. All 17 specimens were identified by Maofa Yang based on their morphological and genital characteristics (Yu, He, and Yang 2021; Yang, Meng, and Li 2017). After species identification, total genomic DNA was extracted from the head and thorax tissues of 17 individual adults using the Tissue and Blood Genome DNA Extraction Kit (Qiagen, Hilden, Germany), following the manufacturer's protocols. The total genomic DNA was finally stored at −20°C for further use, and the external genitalia were preserved in glycerol. Both of them were deposited at the Institute of Entomology, Guizhou University, Guiyang, China (GUGC).

**TABLE 1:** Collection details for 16 newly sequenced species of Mileewinae.

Name	Locality	Collection time	Collector
*Mileewa anchora*	Wuzhi Mountain, Hainan, China	13‐June‐2019	Likun‐Zhong
*Mileewa branchiuma*	Tangjia River, Sichuan, China	19‐August‐2018	Wen‐Zhang
*Mileewa damingana*	Daming Mountain, Guangxi, China	30‐May‐2019	Xiaoli‐Xu
*Mileewa digitata* dy	Motuo County, Xizang, China	3‐July‐2022	Likun‐Zhong
*Mileewa digitata* hy	Motuo County, Xizang, China	30‐June‐2022	Likun‐Zhong
*Mileewa exsertocaputa*	Jingdong County, Yunnan, China	28‐July‐2022	Hongli‐He
*Mileewa gaoligongana*	Xinping County, Yunnan, China	25‐August‐2021	Hongli‐He
*Mileewa holomacula*	Zhujia Mountain, Guizhou, China	7‐April‐2019	Likun‐Zhong
*Mileewa jinpingana*	Fenshuiling National Natural Reserve, Yunnan, China	1‐September‐2021	Yan Jiang
*Mileewa lackstripa*	Shiwan Mountain, Guangxi, China	21‐April‐2019	Yan Jiang
*Mileewa lynchi*	Motuo County, Xizang, China	29‐June‐2022	Hongli‐He
*Mileewa nii*	Fenshuiling National Natural Reserve, Yunnan, China	30‐August‐2021	Ya Hu
*Mileewa polymorpha*	Laojun Mountain, Sichuan, China	16‐June‐2022	Likun‐Zhong
*Mileewa decemspina*	Longling County, Yunnan, China	20‐August‐2021	Yan Jiang
*Mileewa ussurica*	Laojun Mountain, Sichuan, China	16‐June‐2022	Hongli‐He
*Mileewa zhangi*	Wuzhi Mountain, Hainan, China	13‐June‐2019	Likun‐Zhong
*Ujna liangae*	Mengla County, Yunnan, China	9‐August‐2022	Xiaoli‐Xu

### 2.2 Mitogenomes Sequencing, Assembly, and Annotation

The 17 newly sequenced mitochondrial genomes were examined using next‐generation sequencing on the Illumina NovaSeq 6000 platform (Berry Genomics, Beijing, China), employing a paired‐end 150 sequencing approach. This process yielded approximately 6 Gb of clean data for each species. The assembly of purified sequence reads was conducted with GetOrganelle version 1.7.5 (Jin et al. 2020), using the mitochondrial genome sequence of *Mileewa mira* (NC_067805.1) as a reference (He et al. 2022). After that, the 17 mitogenomes were annotated with MitoZ v2.4, employing invertebrate mitochondrial genetic codes (Meng et al. 2019). The annotation results from MitoZ were subsequently imported into Geneious Prime 2021.1 software for further refinement. To improve the precision of 22 tRNAs and 13 PCGs localization, while also constructing secondary structures of tRNAs, we employed the MITOS2 Galaxy tool (The Galaxy Community 2024; Bernt et al. 2013; Donath et al. 2019; Arab et al. 2017), utilizing invertebrate genetic codes. Additionally, open reading frame (ORF) finders within Geneious Prime were applied to annotate PCGs following invertebrate genetic codes standards. The large ribosomal RNA (rrnL) genes were discerned by examining the locations of neighboring tRNA genes (trnL1 and trnV), whereas the small ribosomal RNA (rrnS) genes were identified using predictions from MITOS2 and by comparing them with homologous rrnS genes from other Mileewinae species. Circular maps of mitogenomes were created using the OGDRAW web application (https://chlorobox.mpimp‐golm.mpg.de/OGDraw.html) (Greiner, Lehwark, and Bock 2019).

### 2.3 Mitogenomes Sequence Analysis

The analysis of nucleotide composition and relative synonymous codon usage (RSCU) were conducted using PhyloSuite software version 1.2.3 (Zhang et al. 2020; Xiang et al. 2023). Additionally, the RSCU charts for the 17 mitochondrial genomes were generated using the plotting functions within the software and arranged in Adobe Illustrator CS6. To evaluate strand asymmetry, the formulas AT skew = (A − T)/(A + T) and GC skew = (G − C) / (G + C) were employed (Perna and Kocher 1995). The secondary structures of tRNAs were generated using nc files from MITOS2 and then plotted with VARNAv3‐93 and Rnaviz v2.0.3, with final layout adjustments made in Adobe Illustrator CS6 (Darty, Denise, and Ponty 2009; De Rijk, Wuyts, and De Wachter 2003). Tandem repeats located within the CR were identified through the Tandem Repeats Finder tool (https://tandem.bu.edu/trf/trf.html) (Benson 1999). The nucleotide diversity (Pi) and the ratio of nonsynonymous substitutions (Ka) to synonymous substitutions (Ks) for PCGs were calculated using DNASP v6.0 software (Rozas et al. 2017). The pairwise genetic distances and nucleotide differences derived from PCGs were calculated with the aid of MEGA X (Kumar et al. 2018), utilizing the Kimura 2‐parameter model (Kimura 1980) and the number of differences model, alongside a bootstrap procedure comprising 500 replicates. The 17 newly sequenced mitogenomes of Mileewinae were submitted to GenBank along with a tbl file, utilizing GB2sequin (https://chlorobox.mpimp‐golm.mpg.de/GenBank2Sequin.html) (Lehwark and Greiner 2019), and assigned accession numbers PQ469714‐PQ469730.

### 2.4 Phylogenetic Analysis

Alongside the mitochondrial genomes of the 16 species belonging to the Mileewinae analyzed in this study, all available mitochondrial genome sequences of other species within this subfamily were retrieved from GenBank for the purpose of phylogenetic analysis. In total, 27 species from the Mileewinae were designated as the ingroup, while *Evacanthus heimianus* (MG813486.1) (Wang et al. 2019) and *Evacanthus danmainus* (MN227166.1) from the subfamily Evacanthinae were chosen as the outgroup; comprehensive details can be found in Table 2. In this study, we focused on the analysis of three concatenated data set groups: PCGs, PCGsRNA (13PCGs and 2 rRNAs), and PCGs12RNA (The first and second codon positions of 13 PCGs and 2 rRNAs). The extraction of genes from the 17 mitochondrial genomes was performed using PhyloSuite v1.2.3 (Zhang et al. 2020; Xiang et al. 2023). A total of 13 PCG sequences and two RNA sequences were aligned in batches using MAFFT version 7.471 (Katoh and Standley 2013). The alignment utilized the ‘–auto’ strategy, applying codon alignment mode for the PCGs and normal alignment mode for the RNA sequences. The alignments of the PCGs were subsequently improved with the use of the codon‐aware software MACSE v2.03 (Ranwez et al. 2018). This application maintains the reading frame while accommodating sequencing errors and sequences that exhibit frameshifts. In batches, ambiguously aligned fragments from the alignments of 13 PCGs were eliminated using Gblocks version 0.91b (Talavera and Castresana 2007). The following parameters were applied: a minimum of 16 sequences for a conserved/flank position, a maximum of 8 contiguous non‐conserved positions, a minimum block length of 10, and allowance for gaps in positions (with half). The gap positions in RNA sequences were removed using the trimAl tool (Capella‐Gutiérrez, Silla‐Martínez, and Gabaldón 2009) with the “‐automated1” command. After optimization and trimming, the alignment files for all genes across various data sets were combined using the built‐in functions of PhyloSuite software (Zhang et al. 2020; Xiang et al. 2023). To determine the most suitable partition model and schemes, ModelFinder v2.2.0 (Kalyaanamoorthy et al. 2017) was utilized, employing the corrected Akaike Information Criterion (AICc) for both Bayesian inference (BI) and maximum likelihood (ML) analyses. Detailed models and schemes information is shown in Table S6. Phylogenetic trees based on maximum likelihood were constructed utilizing IQ‐TREE v2.2.0 (Nguyen et al. 2015) with an Edge‐linked partition model, performing 5000 ultrafast bootstraps (Minh, Nguyen, and von Haeseler 2013) to generate bootstrap support (BS) and employing the Shimodaira–Hasegawa–like approximate likelihood ratio test (SH‐aLRT) (Guindon et al. 2010). Phylogenetic trees based on Bayesian Inference were generated using MrBayes v3.2.7a (Ronquist et al. 2012) with partition model that involved four chains (2 parallel runs), comprising 3,115,000 generations for PCGsRNA, 511,000 generations for PCGs12RNA, and 1,145,000 generations for PCGs. The sampling occurred every 1000 generations, and the first 25% of the sampled data were excluded as burn‐in. Once the average standard deviation of split frequencies fell below 0.01 (effective sample size > 200, potential scale reduction factor close to 1), the remaining trees were utilized to create a consensus tree and assess the posterior probability (PP) values. The iTOL online tool (https://itol.embl.de/) (Letunic and Bork 2021) was utilized to visualize and modify phylogenetic trees.

**TABLE 2:** Mitochondrial genomes utilized in the phylogenetic analysis conducted in this research.

Taxon	Accession number	Size (bp)	AT%	References
Evacanthinae (Outgroup)				
*Evacanthus*				
*Evacanthus danmainus*	MN227166.1	15,343	79.4	Unpublished
*Evacanthus heimianus*	MG813486.1	15,806	79.9	Wang et al. (2019)
Mileewinae (Ingroup)				
*Mileewa*				
*Mileewa alara*	MW533151.1	16,020	77.9	He and Yang (2021)
*Mileewa albovittata*	MK138358.1	15,079	79.6	He, Li, and Yang (2019)
*Mileewa amplimacula*	NC_067808.1	15,436	78.6	He et al. (2022)
*Mileewa anchora*	PQ469722	14,842	79.6	This study
*Mileewa branchiuma*	PQ469714	14,888	78.4	This study
*Mileewa damingana*	PQ469730	14,723	79.4	This study
*Mileewa digitata* dy	PQ469728	15,280	80.7	This study
*Mileewa digitata* hy	PQ469726	14,843	80.6	This study
*Mileewa exsertocaputa*	PQ469718	15,009	78.8	This study
*Mileewa gaoligongana*	PQ469727	14,825	79.3	This study
*Mileewa holomacula*	PQ469720	14,888	77.2	This study
*Mileewa jinpingana*	PQ469725	14,586	78.5	This study
*Mileewa lackstripa*	PQ469716	14,839	77.9	This study
*Mileewa lamellata*	NC_067806.1	14,787	80.2	He et al. (2022)
*Mileewa lynchi*	PQ469724	14,839	78.9	This study
*Mileewa margheritae*	MT483998.1	15,375	79	He and Yang (2020b)
*Mileewa mira*	NC_067805.1	14,917	79.1	He et al. (2022)
*Mileewa nii*	PQ469721	14,879	78.7	This study
*Mileewa polymorpha*	PQ469729	14,649	80	This study
*Mileewa ponta*	MT497465.1	15,999	79.9	He and Yang (2020a)
*Mileewa rufivena*	MZ326689.1	15,837	79	Yu and Zhang (2021)
*Mileewa sharpa*	NC_067807.1	14,859	78.4	He et al. (2022)
*Mileewa decemspina*	PQ469719	14,532	79.3	This study
*Mileewa ussurica*	PQ469717	14,815	78.9	This study
*Mileewa zhangi*	PQ469715	14,749	79.8	This study
*Processina*				
*Processina sexmaculata*	NC_067809.1	15,404	78.3	He et al. (2022)
*Ujna*				
*Ujna liangae*	PQ469723	14,564	78.8	This study
*Ujna puerana*	MZ326688.1	14,838	77.1	Yu and Zhang (2021)

### 2.5 Ancestral State Reconstructions Analysis

In our examination of the morphological traits of tribe Mileewini species found in China (Yang, Meng, and Li 2017), we identified significant forewing features necessary for creating a character matrix, which is presented in Table 3. The forewing patch positions traits of these species were categorized as follows: (0) only present on the costal margin; (1) only present on the apical cell; (2) present on both the posterior margin and the apical cell; (3) present on the costal margin, posterior margin, and apical cell; and (4) no patches. The analysis employed a maximum likelihood tree, utilizing the most stable topological structures derived from each data set. Subsequently, we reconstructed the ancestral states for the tribe Mileewini species based on forewing morphology using Mesquite 3.70 software (Maddison and Maddison 2021), applying the “Likelihood Ancestral States” method alongside the Mk1 (est.) model to produce proportional likelihood percentages (PL) for each node. The final reconstructions were then imported into Adobe Illustrator CS6 for layout and editing purposes.

**TABLE 3:** Coding of forewing patch position characteristics.

Subfamily	Genus	Species	Forewing patch position
Evacanthinae	*Evacanthus*	*Evacanthus danmainus*	0
Evacanthinae	*Evacanthus*	*Evacanthus heimianus*	0
Mileewinae	*Mileewa*	*Mileewa alara*	3
Mileewinae	*Mileewa*	*Mileewa albovittata*	1
Mileewinae	*Mileewa*	*Mileewa amplimacula*	2
Mileewinae	*Mileewa*	*Mileewa anchora*	1
Mileewinae	*Mileewa*	*Mileewa branchiuma*	2
Mileewinae	*Mileewa*	*Mileewa damingana*	0
Mileewinae	*Mileewa*	*Mileewa digitata* dy	2
Mileewinae	*Mileewa*	*Mileewa digitata* hy	2
Mileewinae	*Mileewa*	*Mileewa exsertocaputa*	2
Mileewinae	*Mileewa*	*Mileewa gaoligongana*	4
Mileewinae	*Mileewa*	*Mileewa holomacula*	3
Mileewinae	*Mileewa*	*Mileewa jinpingana*	4
Mileewinae	*Mileewa*	*Mileewa lackstripa*	0
Mileewinae	*Mileewa*	*Mileewa lamellata*	2
Mileewinae	*Mileewa*	*Mileewa lynchi*	1
Mileewinae	*Mileewa*	*Mileewa margheritae*	2
Mileewinae	*Mileewa*	*Mileewa mira*	2
Mileewinae	*Mileewa*	*Mileewa nii*	2
Mileewinae	*Mileewa*	*Mileewa polymorpha*	2
Mileewinae	*Mileewa*	*Mileewa ponta*	2
Mileewinae	*Mileewa*	*Mileewa rufivena*	2
Mileewinae	*Mileewa*	*Mileewa sharpa*	0
Mileewinae	*Mileewa*	*Mileewa decemspina*	2
Mileewinae	*Mileewa*	*Mileewa ussurica*	2
Mileewinae	*Mileewa*	*Mileewa zhangi*	0
Mileewinae	*Processina*	*Processina sexmaculata*	3
Mileewinae	*Ujna*	*Ujna liangae*	0
Mileewinae	*Ujna*	*Ujna puerana*	0

## 3 Results

### 3.1 Mitogenomes Organization

The complete mitochondrial genome of *M. digitata* dy measures 15,280 bp in length, whereas the lengths of the other 16 newly sequenced mitochondrial genomes vary from 14,532 bp for *M. decemspina* to 15,009 bp for *M. exsertocaputa* (Figure S1, Tables 2 and S3). All 17 mitochondrial genomes include 13 PCGs, two rRNAs, and 22 tRNAs; however, *M. digitata* dy has one additional CR. Among these genes, 23 (comprising 9 PCGs and 14 tRNAs) are found on the heavy (H) strand, while the light (L) strand contains 14 genes (comprising 4 PCGs, 8 tRNAs, and 2 rRNAs) (Figures S1 and S25, Table S1). The arrangement of genes matches that of previously published mitochondrial genomes within the subfamily Mileewinae (Yu and Zhang 2021; He et al. 2022). The adenine‐thymine (AT) content across the 17 mitochondrial genomes varied between 77.2% for *M. holomacula* and 80.7% for *M. digitata* dy, indicating a notable AT bias (Tables 2 and S3). All 17 mitogenomes displayed a positive AT skew ranging from 0.036 to 0.115, along with a negative GC skew between −0.179 and − 0.058 (Table S3). These results indicate that the quantities of A and C nucleotides exceed those of T and G nucleotides. The analyzed mitochondrial genomes exhibit between 7 and 13 intergenic regions and 10 to 15 overlapping regions (initiating from trnI), with lengths varying from 1 to 37 bp for intergenic regions and from 1 to 10 bp for overlapping regions. The most extensive intergenic region occurs between the trnY and cox1 genes in *M. lackstripa*, while the longest overlap is located between the trnS2 and nad1 genes in *M. gaoligongana*. In addition, comparable intergenic and overlapping regions were observed across the 17 mitochondrial genomes, such as a 3 bp overlap (trnI‐trnQ), an 8 bp overlap (trnW‐trnC), and several 2 bp intergenic regions (nad4L‐trnT; trnP‐nad6), some of which have been noted in earlier research (He et al. 2022). Furthermore, both mitochondrial genomes of *M. digitata* (hy and dy) display complete agreement in the number (15 overlaps and 9 intergenic spacers), position, and dimensions of their intergenic and overlapping regions (Table S2).

### 3.2 Protein‐Coding Genes and Codon Usage Analysis

The total lengths of the 13 PCGs across the 17 mitochondrial genomes vary from 10,914 bp in *M. zhangi* to 10,956 bp in both *M. lynchi* and *M. anchora*. In contrast, the lengths of the 13 PCGs in the mitochondrial genomes of *M. digitata* (dy and hy) are consistently 10,941 bp (Table S3). Among the 13 PCGs, nine genes (cox1, cox2, cox3, atp6, atp8, nad2, nad3, nad6, and cytb) are situated on the H‐strand, while the remaining four genes (nad1, nad4, nad4L, and nad5) are positioned on the L‐strand (Figures S1 and S25, Table S1). Furthermore, among these genes, nad5 is identified as the longest gene while atp8 is the shortest. The overall AT content among the 13 PCGs in the 17 mitochondrial genomes spans from 75.8% to 80%. Additionally, when examining the AT content at various codon positions within the PCGs, it is observed that the third codon position has a higher percentage compared to the first codon position, while the second codon position presents the lowest content. In all mitochondrial genomes, the 13 PCGs display a negative AT skew, with values ranging from −0.171 to −0.124, whereas the GC skew varies between −0.022 and 0.066 (Table S3). A considerable proportion of PCGs begin with the standard codon ATN, while the start codon TTG is present in certain genes, including atp8 and nad5, as noted in previous studies (Yu and Zhang 2021; He et al. 2022). Most PCGs conclude with the complete stop codons TAA and TAG; however, a subset of genes employs an incomplete stop codon labeled T‐ (which may represent TA or T) (Table S2). Such incomplete stop codons are frequently observed in the mitogenomes of invertebrates and can be resolved through posttranscriptional polyadenylation (Wolstenholme 1992; Ojala, Montoya, and Attardi 1981). Through the calculation and statistical analysis of RSCU values alongside the count of codons corresponding to each amino acid (as shown in Figure S2, Table S4), it becomes evident that the codons most frequently utilized include UUU (F), UUA (L), AUU (I), AUA (M), and AAU (N), reflecting a notable AT bias. Additionally, when excluding stop codons, the total number of codons that code for each amino acid in the 13 PCGs across the 17 mitochondrial genomes ranges from 3626 to 3641 (Table S5). Among these, leucine codons are the most prevalent, constituting between 13.31% and 14.57% of the total, followed by isoleucine at 10.96% to 12.72%. In contrast, arginine codons are the least common, accounting for only 1.29% to 1.40% of the total (Figure S3, Table S5).

This study examined the variability and evolutionary rates of the 13 PCGs across the 28 known mitogenomes belonging to the subfamily Mileewinae. For each PCG, we calculated Pi, Ka, Ks, and the Ka/Ks ratios (Figure S4, Table S7). The findings revealed that the overall variability in Pi values was not statistically significant, with values ranging from 0.1529 for cox1 to 0.3158 for atp8. Among these, atp8 demonstrated the highest variability, followed by nad2 with a Pi value of 0.2651 and nad6 at 0.2378. In contrast, cox1 exhibited the lowest variability, signifying it as the most conserved, with a Pi value of 0.1523. The Ka/Ks ratios for the 13 PCGs varied between 0.1069 for cox1 and 0.9063 for atp8, with all values remaining below 1. This suggests that these genes have primarily experienced purifying selection, and they are well‐suited for exploring the phylogenetic relationships present within the subfamily Mileewinae. Among these, atp8 (0.9063), nad2 (0.5160), and nad6 (0.4670) displayed greater evolutionary rates, whereas cox1 represented the lowest evolutionary rate at 0.1069. The findings from the Ka/Ks and Pi analyses indicate that conservative genes, like cox1, which evolve at a slower rate, are particularly well‐suited for creating a barcode database to support traditional taxonomic species identification (Hebert, Ratnasingham, and De Waard 2003; Hebert et al. 2003).

In accordance with the aims of this study—to confirm the presence of polymorphism in *M. digitata* and to offer references for distinguishing species with similar taxonomic features—we assessed the pairwise genetic distances between species by analyzing 13 PCGs along with the cox1 gene obtained from the mitochondrial genomes of 27 species. Additionally, we quantified the nucleotide differences among these species (Tables S8–S11). Analysis of the barcoding gene cox1 indicated that three pairs of species demonstrated genetic distances below 2%: *M. nii* and *M. margheritae* (1.73%), *M. rufivena* and *M. ussurica* (1.45%), and *M. digitata* hy and *M. digitata* dy (1.72%) (Table S8). In contrast, the remaining pairs showed genetic distances exceeding 8.52% (e.g., *M. jinpingana* and *M. gaoligongana*), with nucleotide differences of 26, 22, and 26 across 1536 sites (Table S9). Parallel findings were noted when utilizing the data set of 13 PCGs, which revealed genetic distances of 1.85% between *M. nii* and *M. margheritae*, 1.21% between *M. rufivena* and *M. ussurica*, and 1.61% between *M. digitata* hy and *M. digitata* dy (Table S10). The number of nucleotide differences across 10,968 sites was 198, 130, and 173, respectively (Table S11). A preliminary investigation revealed that intraspecific genetic distances for mitochondrial genes typically do not surpass 2%, with the majority remaining under 1% (Avise 2000). Recent research indicates that a threshold of 2%–3% is frequently applied across the class Insecta; however, around 25% of species exhibit significant intraspecific variation exceeding 3%. Furthermore, when the minimum interspecific genetic distance among congeneric species is at least 2%, it becomes feasible to prevent the overestimation of species diversity based on these empirical thresholds (Zhang and Bu 2022). In the case of subtropical aphids (Hemiptera), a threshold of 2% has proven effective for differentiating most species (Li et al. 2019). Considering the findings from these studies, the genetic distances within the three previously mentioned groups of species remain below 2%, irrespective of whether a threshold of 2% or 3% is employed for species identification. Except for *M. digitata* , which is only recorded in Motuo, the other four species exhibit broad distributions with overlapping ranges. Consequently, considering their comparable morphological traits (Figure S26) (Yang, Meng, and Li 2017), the species examined in this study—*M. nii* and *M. margheritae*, along with *M. rufivena* and *M. ussurica*—may be provisionally classified as belonging to the same species. The existence of polymorphism in *M. digitata* has also been confirmed (Figure 1). Nonetheless, to substantiate this conclusion, it remains essential to sample additional individuals from various collection sites and gather genetic barcode data for a more comprehensive analysis of genetic distances.

### 3.3 Transfer and Ribosomal RNA Genes

The lengths of the 22 tRNA genes spanning all 17 mitochondrial genomes vary from 59 bp (trnA in *M. ussurica* and trnC in *M. zhangi* ) to 72 bp (trnK in all genomes except for *M. exsertocaputa* and *U. liangae*), with a total concatenated length ranging from 1418 bp ( *M. zhangi* and *U. liangae*) to 1449 bp ( *M. digitata* hy) (Tables S1 and S3). Specifically, the total tRNAs length in *M. digitata* dy is 1447 bp. Out of these 22 tRNA genes, 14 are found on the H‐strand, while the remaining 8 are located on the L‐strand (Figures S1 and S25, Table S1). The AT content for these tRNAs across the 17 mitochondrial genomes varies from 79.1% to 82%, with AT skew values between −0.003 and 0.047, and positive GC skew values ranging from 0.114 to 0.185 (Table S3). All tRNAs can adopt a typical cloverleaf secondary structure, except for trnS1 in all species and trnG in *M. damingana*. The former is missing the dihydrouridine (DHU) arm, while the latter lacks the TΨC arm, both of which are substituted by a simple loop. This feature has also been identified in other mitochondrial genomes of the family Cicadellidae (Yu and Zhang 2021; He et al. 2022; Jiang et al. 2021, 2022a; Wang, Wang, and Dai 2021; Lin, Huang, and Zhang 2021; Wang et al. 2020) (Figures S5 and S7–, S22). Furthermore, the anticodons for all tRNAs have been identified among the Cicadellidae species documented in the literature (Yu and Zhang 2021; He et al. 2022; Jiang et al. 2022b; Du et al. 2021; Du, Dietrich, and Dai 2019). The lengths of the DHU and TΨC loops in the predicted secondary structures exhibited variability, leading to variations in the overall lengths of each tRNA. Conversely, the anticodon loop remained highly conserved, measuring 7 bp.

The complete mitochondrial genome of *M. digitata* dy contains two rRNA genes with a total length of 1953 bp. In contrast, the total lengths of the two rRNA genes in the other 16 mitochondrial genomes range from 1952 bp in *M. damingana* to 1981 bp in *M. lynchi* (Table S3). The overall AT content of these genomes fluctuates between 80% and 82%, with AT skew values ranging from −0.175 to −0.101 and positive GC skew values between 0.215 and 0.28 (Table S3). Both rRNA genes are situated on the L‐strand, where the rrnL gene is located between trnL1 and trnV, exhibiting lengths from 1195 bp in *M. anchora* and *M. polymorpha* to 1224 bp in *M. lynchi* (Figures S1 and S25, Tables S1 and S3). The rrnS gene was annotated and corrected through MITOS2, alongside comparisons with reference homologous species, featuring lengths that vary from 743 bp in *M. damingana* to 758 bp in *M. polymorpha* , *M. exsertocaputa*, and *M. anchora*. In *M. digitata* dy, the rrnL and rrnS genes measure 1202 bp and 751 bp, respectively.

### 3.4 Control Region

The mitochondrial control region of *M. digitata* dy measures 949 bp in length, which can be categorized as moderate when compared to other published control regions within the subfamily Mileewinae (Yu and Zhang 2021; He et al. 2022) (Tables S1 and S3). This region is situated between the rrnS and trnI genes and exhibits an AT content of 86.4%, surpassing that of tRNAs, rRNAs, and PCGs (Figure S1, Tables S1 and S3). The AT skew and GC skew values for this region are both positive, recorded at 0.134 and 0.24, respectively (Table S3). Furthermore, it harbors a repetitive unit consisting of 59 bp, which is repeated 11 times (11 × 59 bp) (Figure S6). Our analysis revealed the existence of a 19 bp poly(A) stretch at the beginning of the control region. This finding is consistent with earlier studies that identified similar stretches in the control regions of *M. amplimacula* (21 bp), *P. sexmaculata* (20 bp), and *M. lamellata* (20 bp) (He et al. 2022).

### 3.5 Phylogenetic Analysis

**FIGURE 2:**
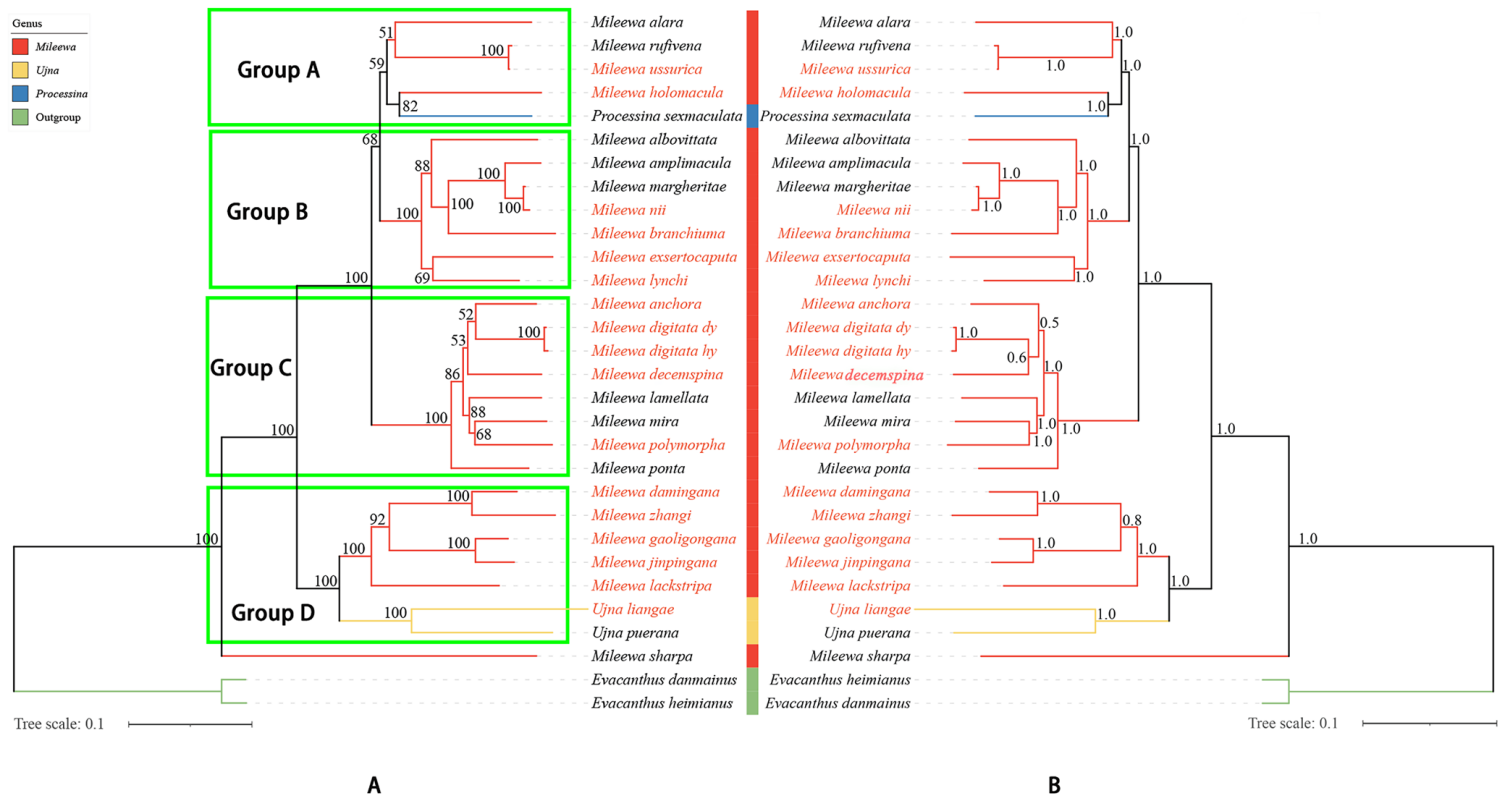
Phylogenetic tree inferred from the PCGs12RNA data set using Maximum Likelihood (ML) and Bayesian Inference (BI) methods. Bootstrap support (BS) and posterior probability (PP) values are displayed at the nodes of the trees. Mitogenomes that were newly sequenced are highlighted in red. The topological structures of Groups A‐D are highlighted within the green box. (A) Maximum Likelihood (ML) tree constructed from the PCGs12RNA data set. (B) Bayesian Inference (BI) tree generated from the PCGs12RNA data set. PCGs12RNA: The first and second codon positions of 13 protein‐coding genes (PCGs) and 2 ribosomal RNA genes (rRNAs).

This study builds on prior mitochondrial genome research, which confirmed the monophyly of the subfamily Mileewinae (Yu and Zhang 2021; He et al. 2022). By integrating 17 newly sequenced mitochondrial genomes, the investigation aims to elucidate the phylogenetic relationships among genera and species of the tribe Mileewini in China. The findings are expected to offer fresh insights and molecular support for the classification of certain similar species and links at the genus level, enhancing existing traditional taxonomic frameworks. In this study, phylogenetic trees were constructed using 29 species (Table 2), leveraging both BI and ML methods. The ingroup comprised 27 species from the subfamily Mileewinae, which included 17 newly published mitochondrial genomes from this research. Conversely, the outgroup consisted of two species from the subfamily Evacanthinae sourced from GenBank. The reconstruction process involved six phylogenetic trees derived from three distinct data sets, which were based on 13 PCGs and 2 rRNAs (Figures 2, S23, and S24). These data sets were labeled as PCGs, PCGsRNA, and PCGs12RNA.

The topological frameworks of these six trees show variability; nonetheless, they reveal overarching similarities in their phylogenetic relationships. To aid clarity, these similar topological structures can be divided into four distinct groups (Groups A‐D) (Figure 2A). Group A encompasses five trees exhibiting consistent topological arrangements: PCGs_ML, PCGs_BI (Figure S24), PCGsRNA_ML (Figure S23A), PCGs12RNA_ML, and PCGs12RNA_BI (Figure 2) characterized by the arrangement (BS > 59, PP = 1): ((*M. alara* + (*M. rufivena* + *M. ussurica*)) + (*M. holomacula* + *P. sexmaculata* )). Group B demonstrates uniform topological structures in PCGsRNA_ML, PCGsRNA_BI (Figure S23), PCGs12RNA_ML, and PCGs12RNA_BI (Figure 2), represented as (BS = 100, PP = 1): (( *M. albovittata* + ((*M. amplimacula* + (*M. margheritae* + *M. nii*)) + *M. branchiuma*)) + (*M. exsertocaputa* + *M. lynchi*)). Group C maintains identical topological configurations across the trees PCGs_ML, PCGs_BI (Figure S24), PCGsRNA_ML, PCGsRNA_BI (Figure S23), and PCGs12RNA_ML (Figure 2A), structured as (BS = 100, PP = 1): ((((*M. anchora* + ( *M. digitata* dy + *M. digitata* hy)) + *M. decemspina*) + ( *M. lamellata* + ( *M. mira* + *M. polymorpha* ))) + *M. ponta* ). In Group D, consistent topological structures are observed across PCGs_ML, PCGs_BI (Figure S24), PCGsRNA_ML (Figure S23A), PCGs12RNA_ML, and PCGs12RNA_BI (Figure 2), represented by (BS = 100, PP = 1): ((((*M. damingana* + *M. zhangi* ) + (*M. gaoligongana* + *M. jinpingana*)) + *M. lackstripa*) + (*U. liangae* + *U. puerana*)). Furthermore, *M. sharpa* is consistently located at the base of the phylogenetic trees in all six tree representations (Figures 2, S23, and S24).

The phylogenetic analysis of the six trees indicates that the three genera from the tribe Mileewini in China—*Mileewa*, *Ujna*, and *Processina*—do not exhibit monophyly at the genus level. Within Group D, the two species belonging to the genus *Ujna*, namely, *U. liangae* and *U. puerana*, displayed a strong sister relationship and consistently clustered with five other species from the genus *Mileewa* (BS = 100, PP = 1) (Figures 2, S23, and S24). In Group A, the *Processina* species *P. sexmaculata* was consistently grouped with *M. holomacula* across all examined trees (BS > 56, PP > 0.9) (Figures 2, S23, and S24), reinforcing our earlier results (He et al. 2022). In terms of interspecific relationships, stable clusters with high support (BS = 100, PP = 1) were observed among *M. digitata* hy and *M. digitata* dy, *M. nii* and *M. margheritae*, as well as *M. ussurica* and *M. rufivena*, confirming their minor distinctions and similarities (Figures 1, 2, S23, S24, and S26). Nonetheless, since there exists only one mitochondrial genome data set for *Processina* and limited information for *Ujna*, it is imperative to expand the mitochondrial genome data for these two genera to further investigate the phylogenetic connections within the tribe Mileewini.

### 3.6 Ancestral State Reconstructions of Forewing

**FIGURE 3:**
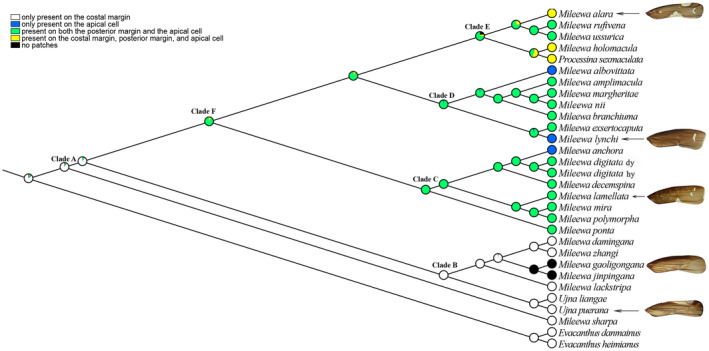
Reconstruction of ancestral states of forewing patch positions based on Maximum Likelihood (ML) method.

Ancestral state reconstruction is an essential technique in evolutionary biology that facilitates the inference of traits in extinct species and enhances our understanding of trait evolution (Meade and Pagel 2022; Omland 1999). The ancestral state reconstruction utilized the ML tree derived from the PCGs12RNA data set, as its topological structure is considered the most representative. The results of the ancestral state reconstruction based on forewing patch positions in this study are illustrated in Figure 3. These findings suggest that the common ancestral state for forewing patch positions within the tribe Mileewini in China has been determined to be only present on the costal margin (Clade A, PL = 92). Furthermore, the forewing patch positions have experienced multiple transitions among the various Mileewini species. At the base of the phylogenetic tree, *M. gaoligongana* and *M. jingpingana* exhibit a change from only present on the costal margin to no patches, while the most recent common ancestors of Clade B and *M. sharpa* remain aligned with the common ancestor of the tribe Mileewini (PL = 99). In Clades C and D, *M. anchora*, *M. lynchi*, and *M. albovittata* shifted from only present on the costal margin to only present on the apical cell, while other species transitioned to present on both the posterior margin and the apical cell. The most recent common ancestors in both Clade C and Clade D are categorized as present on both the posterior margin and the apical cell, with PL values of 99 and 96, respectively. Additionally, part of Clade E at the top of the phylogenetic tree evolved from only present on the costal margin to present on both the posterior margin and the apical cell, whereas another part transitioned to present on the costal margin, posterior margin, and apical cell. Their most recent common ancestor is also characterized as present on both the posterior margin and the apical cell (PL = 75). Furthermore, the most recent common ancestors across Clade C, D, and E consistently show present on both the posterior margin and apical cell (Clade F, PL = 90). Overall, the forewing patch positions in the tribe Mileewini of China demonstrate a transition from being concentrated on the costal margin to encompassing the posterior margin, reflecting a shift from a simpler to a more complex arrangement of patches.

## 4 Discussion

The subfamily Mileewinae consists of around 160 species worldwide, with 71 identified in China (Yu and Zhang 2022; Yan et al. 2021; He et al. 2021; He, Yan, and Yang 2021; Yu, He, and Yang 2021; Yang, Meng, and Li 2017). Despite this diversity, only 11 mitochondrial genomes are accessible in GenBank (Yu and Zhang 2021; He et al. 2022; He and Yang 2020a, 2020b, 2021; He, Li, and Yang 2019), highlighting a significant gap in genomic data. This study presents the sequencing and analysis of 17 mitochondrial genomes of Mileewinae, marking the first report of these genomes. A comparative analysis was also performed on the polymorphism of the unique species *M. digitata* from Motuo, Xizang, in relation to similar species classified using traditional morphological methods. The lengths of the 17 mitochondrial genomes ranged from 14,532 bp for *M. decemspina* to 15,280 bp for *M. digitata* dy, each comprising 13 PCGs, 22 tRNAs, and 2 rRNAs; notably, *M. digitata* dy includes a CR. The gene arrangement observed was consistent with those documented in other Mileewinae mitochondrial genomes (Yu and Zhang 2021; He et al. 2022; He and Yang 2020a, 2020b, 2021; He, Li, and Yang 2019). The overall AT content across these genomes varied between 77.2% and 80.7%, with a positive AT skew ranging from 0.036 to 0.115 and a negative GC skew from −0.179 to −0.058, which corresponds with findings from previously studied Mileewinae genomes (He et al. 2022). Within the mitochondrial genome of *M. digitata* dy, the CR exhibited the highest AT content (86.4%), while the PCGs recorded the lowest (79.9%), following this order of abundance: CR > rRNAs > tRNAs > PCGs. For the PCGs, the AT content hierarchy, from highest to lowest, was: third position (93.6%), first position (75.5%), and second position (70.8%), with similar patterns evident in the other 16 genomes examined in this study and previous work (He et al. 2022). Additionally, the 17 mitochondrial genomes were found to contain between 7 and 13 intergenic regions (1–37 bp) and 10 to 15 overlapping regions (1–10 bp). In contrast, earlier studies investigating five Mileewinae species reported between 10 and 16 overlapping regions (1–12 bp) and 5 to 14 intergenic regions (1–24 bp) (He et al. 2022). This study also identifies a 2 bp interval between nad4L and trnT, as well as between trnP and nad6. Additionally, there are overlapping regions of 8 bp between trnW and trnC, and 3 bp between trnI and trnQ. These features are characteristic of Mileewinae mitochondrial genomes. Notably, the mitochondrial genomes of *M. digitata* (specimens dy and hy) demonstrated complete congruence in terms of quantity, location, and size. The ORFs of the 17 mitochondrial PCGs vary in length from 10,914 bp to 10,956 bp, with both *M. digitata* hy and *M. digitata* dy recorded at 10,941 bp. Most of the PCGs begin with the ATN codon, although atp8 and nad5 start with TTG. While the majority of the genes conclude with the stop codons TAA or TAG—specifically, all atp6 and nad4L genes end with TAA—there are a few exceptions where certain genes terminate with the incomplete stop codons T or TA. All tRNAs display a typical cloverleaf configuration, with the exception of trnS1, which is missing a DHU arm, and trnG in *M. damingana*, which lacks a TΨC arm. This finding aligns with observations from other mitochondrial genomes within the Mileewinae (Yu and Zhang 2021; He et al. 2022). Analysis of the Pi and ka/ks ratios derived from the PCGs indicates that atp8 exhibits the greatest variability and highest evolutionary rate, whereas cox1 is characterized by the lowest evolutionary rate and minimal variability, corroborating findings from previous research (Yu and Zhang 2021). This suggests that PCGs, particularly cox1, are effective for species identification purposes (Hebert, Ratnasingham, and De Waard 2003; Hebert et al. 2003). Consequently, we calculated the genetic distances among the 27 species of Mileewinae currently known, focusing on cox1 and the PCGs. The results show that the genetic distances between *M. nii* and *M. margheritae*, *M. rufivena*, and *M. ussurica*, as well as *M. digitata* hy and *M. digitata* dy, are all below 2%, with slight variation in the number of loci examined. Drawing from earlier research on genetic distance thresholds and their classification attributes (Figure S26) (Zhang and Bu 2022; Li et al. 2019), this study provisionally classifies the species pairs *M. nii* and *M. margheritae*, as well as *M. rufivena* and *M. ussurica*, as belonging to the same species. Simultaneously, molecular data analysis has validated the intraspecific differences observed among male individuals of the species *M. digitata* (Figure 1). In this study, the mitochondrial genome's CR of *M. digitata* dy measures 949 bp, a length deemed moderate relative to other mitochondrial genomes in the subfamily Mileewinae (Yu and Zhang 2021; He et al. 2022). This CR includes a 59 bp repeat unit that appears 11 times. Furthermore, a 19 bp poly A stretch was observed at the beginning of the control region; analogous features have been reported in the mitochondrial genomes of *M. amplimacula* (21 bp), *P. sexmaculata* (20 bp), and *M. lamellata* (20 bp) (He et al. 2022).

Utilizing three data sets—PCGs, PCGsRNA, and PCGs12RNA—we developed six phylogenetic trees through both BI and ML methods. The support rate for the BI trees was found to be marginally superior to that of the ML trees. A comparative analysis of the tree topologies revealed that none of the trees supported the monophyly of the three genera—*Mileewa*, *Processina*, and *Ujna*—within the tribe Mileewini. Despite incorporating 17 new mitochondrial genomes of Mileewinae for phylogenetic investigation in the tribe Mileewini, our findings were consistent with earlier research (He et al. 2022), highlighting inconsistencies with the current morphological classification system. The traditional characteristics used to differentiate these three genera appear inadequate for explaining our phylogenetic results. Notably, the genetic distance results from the PCGs and cox1 gene analyses were confirmed by the phylogenetic trees, where *M. digitata* hy and *M. digitata* dy, *M. nii* and *M. margheritae*, and *M. ussurica* and *M. rufivena* were grouped with strong support (BS = 100, PP = 1), indicating their close similarities in body color, size, and male external genitalia (Figures 1 and S26) (Yu, He, and Yang 2021; Yang, Meng, and Li 2017). Nevertheless, factors such as geographic isolation and ecological adaptation necessitate extensive sampling and genetic data collection from various individuals to substantiate our findings. Importantly, *M. sharpa* consistently appeared at the base of the six phylogenetic trees, suggesting it is more ancestral than the other species in the tribe Mileewini. Based on the insights from these six phylogenetic trees, the current phylogenetic structure of the subfamily Mileewinae in China can be articulated as: ((((((*M. alara* + (*M. rufivena* + *M. ussurica*)) + (*M. holomacula* + *P. sexmaculata* )) + (( *M. albovittata* + ((*M. amplimacula* + (*M. margheritae* + *M. nii*)) + *M. branchiuma*)) + (*M. exsertocaputa* + *M. lynchi*))) + ((((*M. anchora* + ( *M. digitata* dy + *M. digitata* hy)) + *M. decemspina*) + ( *M. lamellata* + ( *M. mira* + *M. polymorpha* ))) + *M. ponta* )) + ((((*M. damingana* + *M. zhangi* ) + (*M. gaoligongana* + *M. jinpingana*)) + *M. lackstripa*) + (*U. liangae* + *U. puerana*))) + *M. sharpa*). Furthermore, it is crucial to expand the mitochondrial genome database for the tribe Mileewini in China to enhance the validation of our phylogenetic findings and to investigate the inter‐generic phylogenetic relationships.

Following the establishment of phylogenetic relationships among the Chinese Mileewini species, we employed the ML tree derived from the PCGs12RNA data set to map the characteristics of forewing patch positions onto the tree. This approach facilitated the reconstruction of ancestral states associated with these positions. Our findings indicated that the common ancestor of forewing patch positions within this tribe was exclusively located on the costal margin. The basal species, *M. sharpa*, along with those in Clade B, predominantly retained this ancestral state, while species in Clade F exhibited a transition to a mixed distribution of patch positions on the apical cell, posterior margin, and costal margin. The ancestral states of forewing patches for the entire Mileewini tribe evolved from being limited to the costal margin to also include patches on the posterior margin and apical cell, before re‐emerging on the costal margin within the most evolutionarily advanced group at the tree's terminal end. Our analysis of forewing patch ancestral states suggests that the extant Chinese Mileewini species can be categorized into two groups (genera): one group has forewing patches only on the costal margin (with the absence of patches regarded as a derived trait), and the other group features patches on both the apical cell and the posterior margin (considering patches solely on the apical cell as a derived trait). Considering both previous and current molecular phylogenetic studies alongside morphological patterns of forewing patches (Yu and Zhang 2021; He et al. 2022; Yang, Meng, and Li 2017), we propose this classification system as the most effective integration of these elements. Nonetheless, the absence of species from the genus *Anzihelus* and the limited data on *Ujna* and *Processina* necessitate further molecular evidence to validate our proposed classification framework in the future.

## 5 Conclusions

This research seeks to investigate the phylogenetic relationships among the species of the subfamily Mileewinae located in China, confirm the monophyletic status of the genera, and provide a molecular foundation for distinguishing between morphologically similar species. To achieve this, a comprehensive analysis of mitochondrial genomes was performed on various morphological individuals of the endemic species *M. digitata* , collected from Motuo, Xizang, as well as on 15 other species from the genera *Mileewa* and *Ujna*. The mitochondrial genome lengths ranged from 14,532 to 15,280 bp, revealing similarities in genomic composition, arrangement of genes, variability in PCGs, evolutionary rates, and secondary structures of tRNAs when compared to previously documented Mileewinae species. Moreover, analyses of genetic distances demonstrated that the differences among two traditionally recognized groups of similar species (*M. nii* and *M. margheritae*, *M. rufivena* and *M. ussurica*), as well as the two morphs of *M. digitata* , were all less than 2%. Considering the morphological similarities and comparable traits in male external genitalia across these groups, we tentatively classify these two pairs as belonging to the same species. Additionally, the observation of polymorphism in *M. digitata* has been substantiated.

This research contributed to the molecular database for the subfamily Mileewinae by sequencing 17 mitochondrial genomes. The comparative analysis of these genomes improved understanding of the structural characteristics of mitochondrial genomes within this group. Additionally, constructing six phylogenetic trees—using both the newly sequenced data and existing mitochondrial genome information for Chinese Mileewinae—further challenged the monophyly of the genera *Ujna*, *Processina*, and *Mileewa*. Moreover, we reconstructed the ancestral state of the tribe Mileewini in China, focusing on the forewing patch position, which offers a fresh perspective on the evolutionary dynamics of this trait among the genera and species in the subfamily. Based on these reconstruction results, we proposed a novel classification framework that categorizes Chinese Mileewini species into two groups. Nevertheless, the new classification system will require validation through phylogenetic results derived from additional mitochondrial genome data in the future. The findings presented in this study provide important insights and direction for the classification and phylogenetic research of the subfamily Mileewinae in the future.

## Author Contributions

**Hongli He:** data curation (equal), formal analysis (equal), methodology (equal), software (equal), visualization (equal), writing – original draft (lead), writing – review and editing (equal). **Bin Yan:** conceptualization (equal), funding acquisition (equal), investigation (equal), writing – review and editing (equal). **Xiaofei Yu:** funding acquisition (equal), investigation (equal), resources (equal), writing – review and editing (equal). **Maofa Yang:** conceptualization (equal), funding acquisition (equal), project administration (equal), supervision (equal), writing – review and editing (equal).

## Conflicts of Interest

The authors declare no conflicts of interest.

## Supporting information


**Figure S1.** Circular representation of the mitochondrial genome of *Mileewa digitata* dy. Genes are depicted using distinct color blocks. Color blocks that appear outside the circle signify the presence of genes on the heavy strand (H‐strand), while those found inside the circle represent genes associated with the light strand (L‐strand).

**Figure S2.** Relative synonymous codon usage (RSCU) values for protein‐coding genes (PCGs) from the 17 newly sequenced mitogenomes of Mileewinae.

**Figure S3.** The distribution of codon counts for each amino acid across the protein‐coding genes (PCGs) in 17 newly sequenced mitochondrial genomes of Mileewiane.

**Figure S4.** Values of nucleotide diversity (Pi) and the ratio of nonsynonymous substitutions (Ka) to synonymous substitutions (Ks) for protein‐coding genes (PCGs) in the mitochondrial genomes of 27 Mileewinae species.

**Figure S5.** Forecasted secondary structures of the 22 transfer RNAs (tRNAs) found in the mitogenome of *Mileewa digitata* dy.

**Figure S6.** Structure of the control region in the mitogenome of *Mileewa digitata* dy. Various repetitive sequences within the tandem repeat unit are denoted by distinct colors, while an asterisk (*) indicates a mismatch.

**Figure S7.** Forecasted secondary structures of the 22 transfer RNAs (tRNAs) found in the mitogenome of *Mileewa digitata* hy.

**Figure S8.** Forecasted secondary structures of the 22 transfer RNAs (tRNAs) found in the mitogenome of *Mileewa anchora*.

**Figure S9.** Forecasted secondary structures of the 22 transfer RNAs (tRNAs) found in the mitogenome of *Mileewa branchiuma*.

**Figure S10.** Forecasted secondary structures of the 22 transfer RNAs (tRNAs) found in the mitogenome of *Mileewa damingana*.

**Figure S11.** Forecasted secondary structures of the 22 transfer RNAs (tRNAs) found in the mitogenome of *Mileewa exsertocaputa*.

**Figure S12.** Forecasted secondary structures of the 22 transfer RNAs (tRNAs) found in the mitogenome of *Mileewa gaoligongana*.

**Figure S13.** Forecasted secondary structures of the 22 transfer RNAs (tRNAs) found in the mitogenome of *Mileewa holomacula*.

**Figure S14.** Forecasted secondary structures of the 22 transfer RNAs (tRNAs) found in the mitogenome of *Mileewa jinpingana*.

**Figure S15.** Forecasted secondary structures of the 22 transfer RNAs (tRNAs) found in the mitogenome of *Mileewa lackstripa*.

**Figure S16.** Forecasted secondary structures of the 22 transfer RNAs (tRNAs) found in the mitogenome of *Mileewa lynchi*.

**Figure S17.** Forecasted secondary structures of the 22 transfer RNAs (tRNAs) found in the mitogenome of *Mileewa nii*.

**Figure S18.** Forecasted secondary structures of the 22 transfer RNAs (tRNAs) found in the mitogenome of *Mileewa polymorpha*.

**Figure S19.** Forecasted secondary structures of the 22 transfer RNAs (tRNAs) found in the mitogenome of *Mileewa decemspina*.

**Figure S20.** Forecasted secondary structures of the 22 transfer RNAs (tRNAs) found in the mitogenome of *Mileewa ussurica*.

**Figure S21.** Forecasted secondary structures of the 22 transfer RNAs (tRNAs) found in the mitogenome of *Mileewa zhangi*.

**Figure S22.** Forecasted secondary structures of the 22 transfer RNAs (tRNAs) found in the mitogenome of *Ujna liangae*.

**Figure S23.** Phylogenetic tree inferred from the PCGsRNA data set using Maximum Likelihood (ML) and Bayesian Inference (BI) methods. Bootstrap support (BS) and posterior probability (PP) values are displayed at the nodes of the trees. Mitogenomes that were newly sequenced are highlighted in red. (A) Maximum likelihood (ML) tree constructed from the PCGsRNA data set. (B) Bayesian inference (BI) tree generated from the PCGsRNA data set. PCGsRNA: 13 protein‐coding genes (PCGs) and 2 ribosomal RNA genes (rRNAs).

**Figure S24.** Phylogenetic tree inferred from the PCGs data set using Maximum Likelihood (ML) and Bayesian Inference (BI) methods. Bootstrap support (BS) and posterior probability (PP) values are displayed at the nodes of the trees. Mitogenomes that were newly sequenced are highlighted in red. (A) Maximum Likelihood (ML) tree constructed from the PCGs data set. (B) Bayesian inference (BI) tree generated from the PCGs data set. PCGs: 13 protein‐coding genes (PCGs).

**Figure S25.** Gene arrangement of 16 newly sequenced mitochondrial genomes of Mileewinae species. The rectangular blocks indicating the genes point right or left to denote their location on the heavy strand (H‐strand) and light strand (L‐strand), respectively.

**Figure S26.** Two pairs of morphologically similar species in this study. A, *Mileewa rufivena.* B, *Mileewa ussurica*. C, *Mileewa Margheritae*. D, *Mileewa nii*.

**TABLE S1**. Structural organization of the mitochondrial genomes in 16 newly sequenced species of Mileewinae.
**TABLE S2**. Start/stop codons and intergenic nucleotides in protein‐coding genes (PCGs) among 17 newly sequenced Mileewinae mitochondrial genomes.
**TABLE S3**. Composition of nucleotides in the mitogenomes of 16 newly sequenced Mileewinae species.
**TABLE S4**. Codon count and the relative synonymous codon usage (RSCU) values for protein‐coding genes (PCGs) in the mitogenomes of 16 newly sequenced Mileewinae species.
**TABLE S5**. The quantity and relative frequencies of codons corresponding to each amino acid within the protein‐coding genes (PCGs) of the 17 newly sequenced mitogenomes of Mileewinae.
**TABLE S6**. Optimal partitioning strategies and substitution models employed in Bayesian Inference (BI) and Maximum Likelihood (ML) phylogenetic analyses.
**TABLE S7**. The nonsynonymous substitutions (Ka) to synonymous substitutions (Ks) and nucleotide diversity (Pi) values calculated from the protein‐coding genes (PCGs) of the mitochondrial genomes of 27 Mileewinae species.
**TABLE S8**. Pairwise genetic distances of 27 Mileewinae species calculated based on the cox1 gene using the Kimura 2‐parameter model.
**TABLE S9**. Pairwise genetic distances of 27 Mileewinae species calculated based on the cox1 gene using the No. of differences model.
**TABLE S10**. Pairwise genetic distances of 27 Mileewinae species calculated based on 13 protein‐coding genes (PCGs) using the Kimura 2‐parameter model.
**TABLE S11**. Pairwise genetic distances of 27 Mileewinae species calculated based on 13 protein‐coding genes (PCGs) using the No. of differences model.

## Data Availability

The mitochondrial DNA sequences for 16 newly sequenced species belonging to the genera *Mileewa* and *Ujna* can be found in GenBank with accession number: PQ469714‐PQ469730.
