# Supplementary figures and images for: Comparative Analysis of Seventeen Mitochondrial Genomes of Mileewinae Leafhoppers, Including the Unique Species Mileewa digitata (Hemiptera: Cicadellidae: Mileewinae) From Xizang, China, and New Insights Into Phylogenetic Relationships Within Mileewini
Source: Ecol Evol. 2025 Jan 9;15(1):e70830. doi: 10.1002/ece3.70830 (PMC11718102; doi:10.1002/ece3.70830)

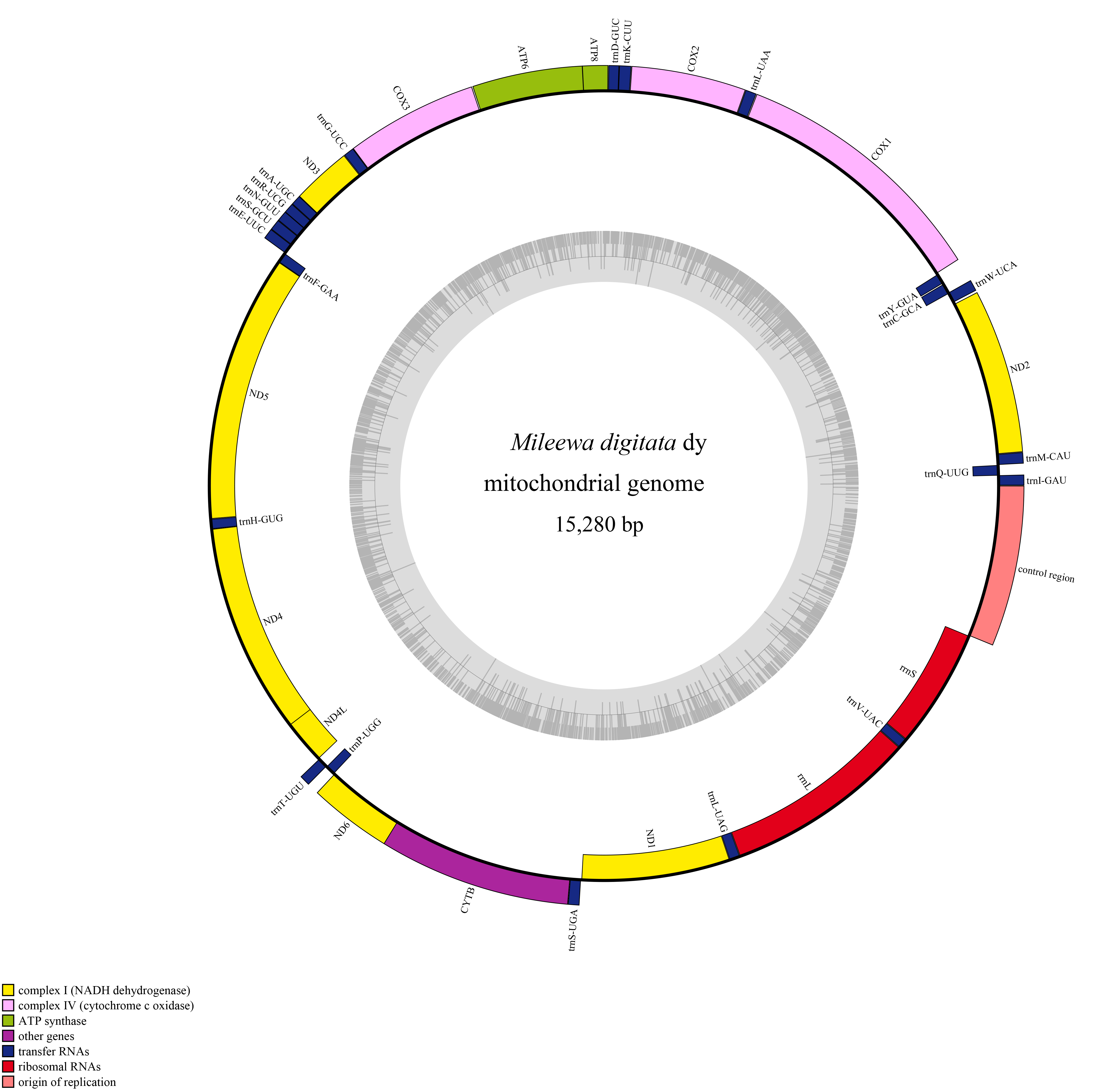

Supplement: Supplementary file 1 — Figure S1. Circular representation of the mitochondrial genome of Mileewa digitata dy. Genes are depicted using distinct color blocks. Color blocks that appear outside the circle signify the presence of genes on the heavy strand (H‐strand), while those found inside the circle represent genes associated with the light strand (L‐strand). [file ECE3-15-e70830-s001.tif]

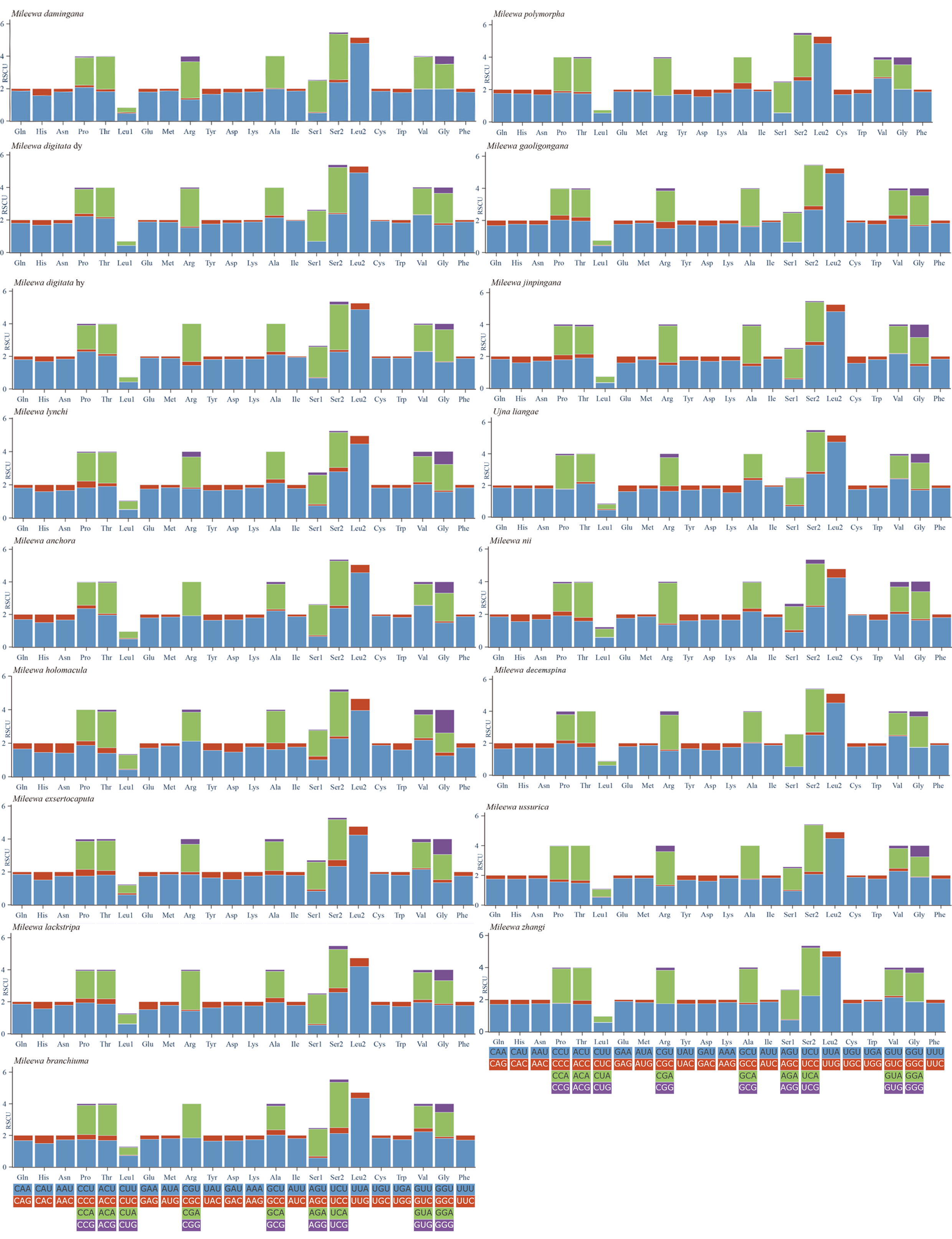

Supplement: Supplementary file 2 — Figure S2. Relative synonymous codon usage (RSCU) values for protein‐coding genes (PCGs) from the 17 newly sequenced mitogenomes of Mileewinae. [file ECE3-15-e70830-s005.tif]

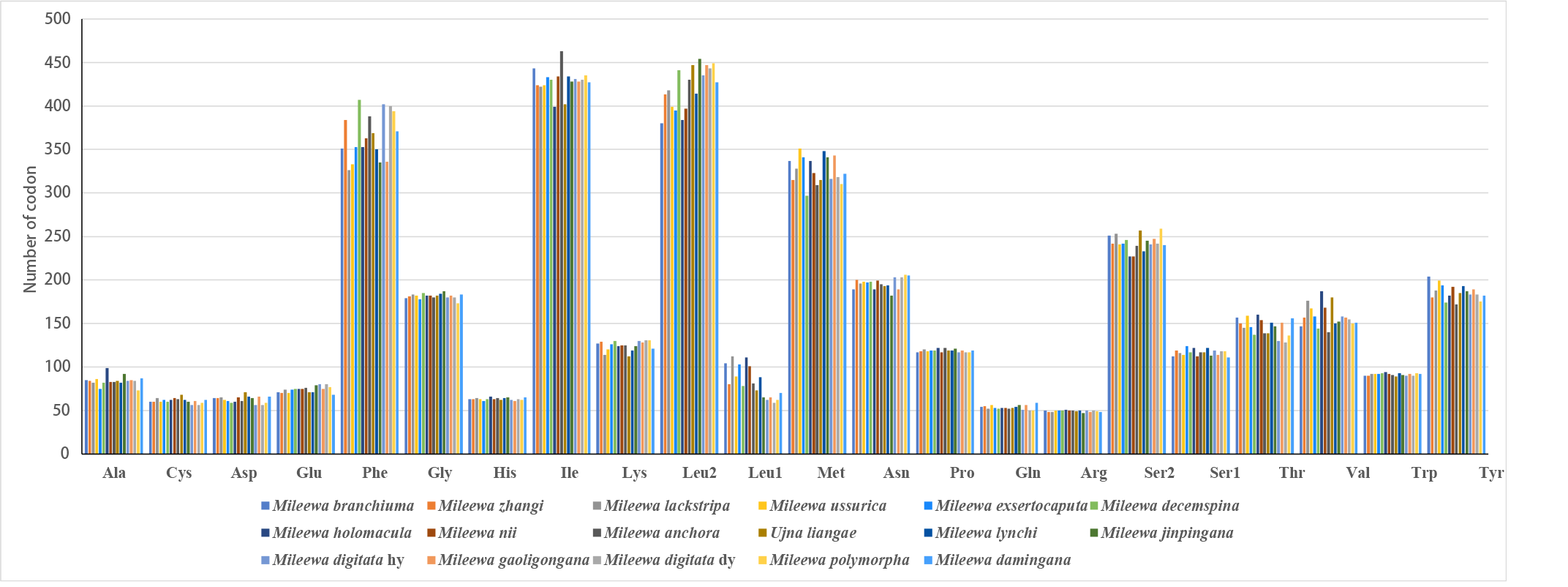

Supplement: Supplementary file 3 — Figure S3. The distribution of codon counts for each amino acid across the protein‐coding genes (PCGs) in 17 newly sequenced mitochondrial genomes of Mileewiane. [file ECE3-15-e70830-s022.tif]

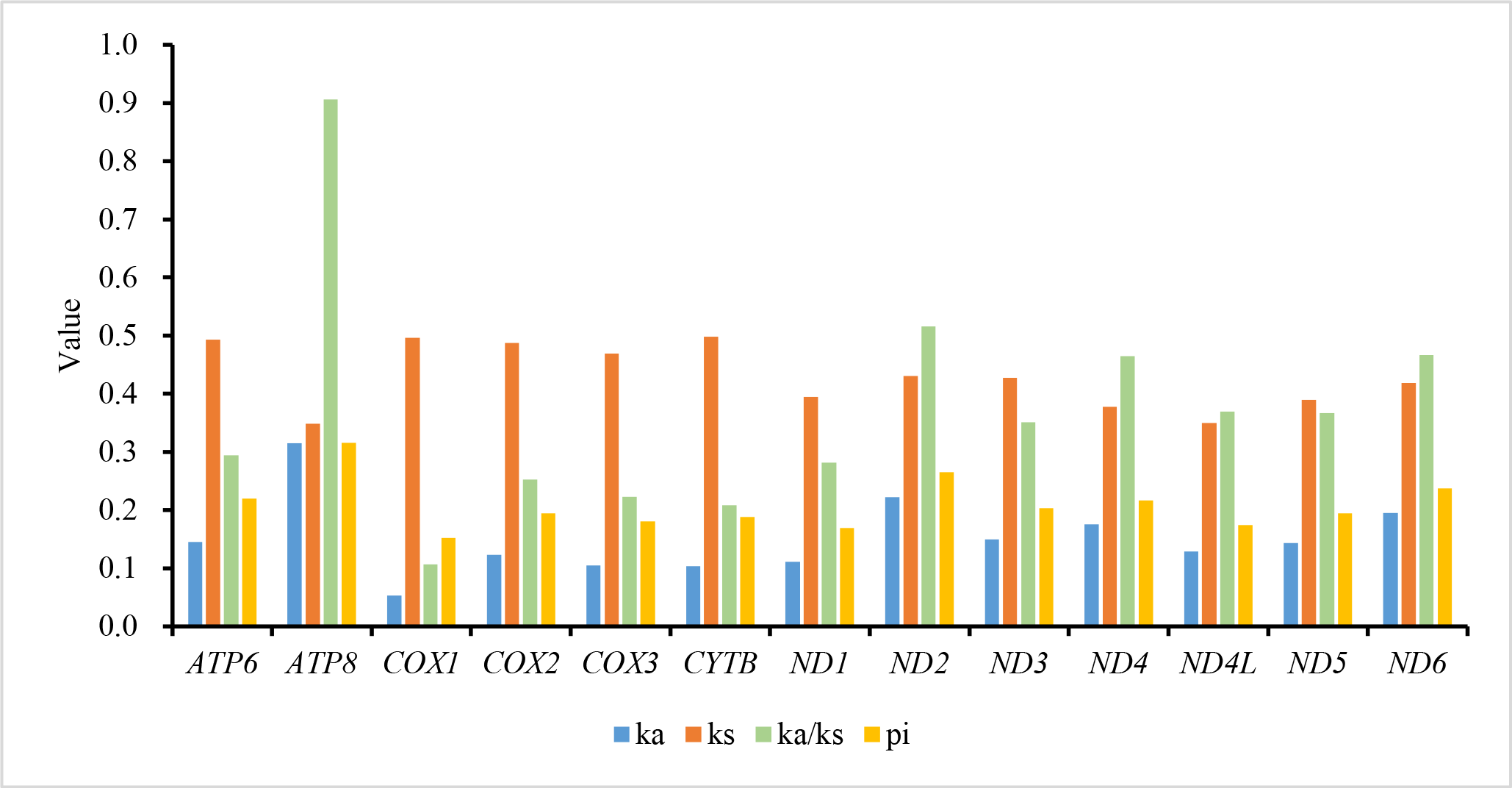

Supplement: Supplementary file 4 — Figure S4. Values of nucleotide diversity (Pi) and the ratio of nonsynonymous substitutions (Ka) to synonymous substitutions (Ks) for protein‐coding genes (PCGs) in the mitochondrial genomes of 27 Mileewinae species. [file ECE3-15-e70830-s013.tif]

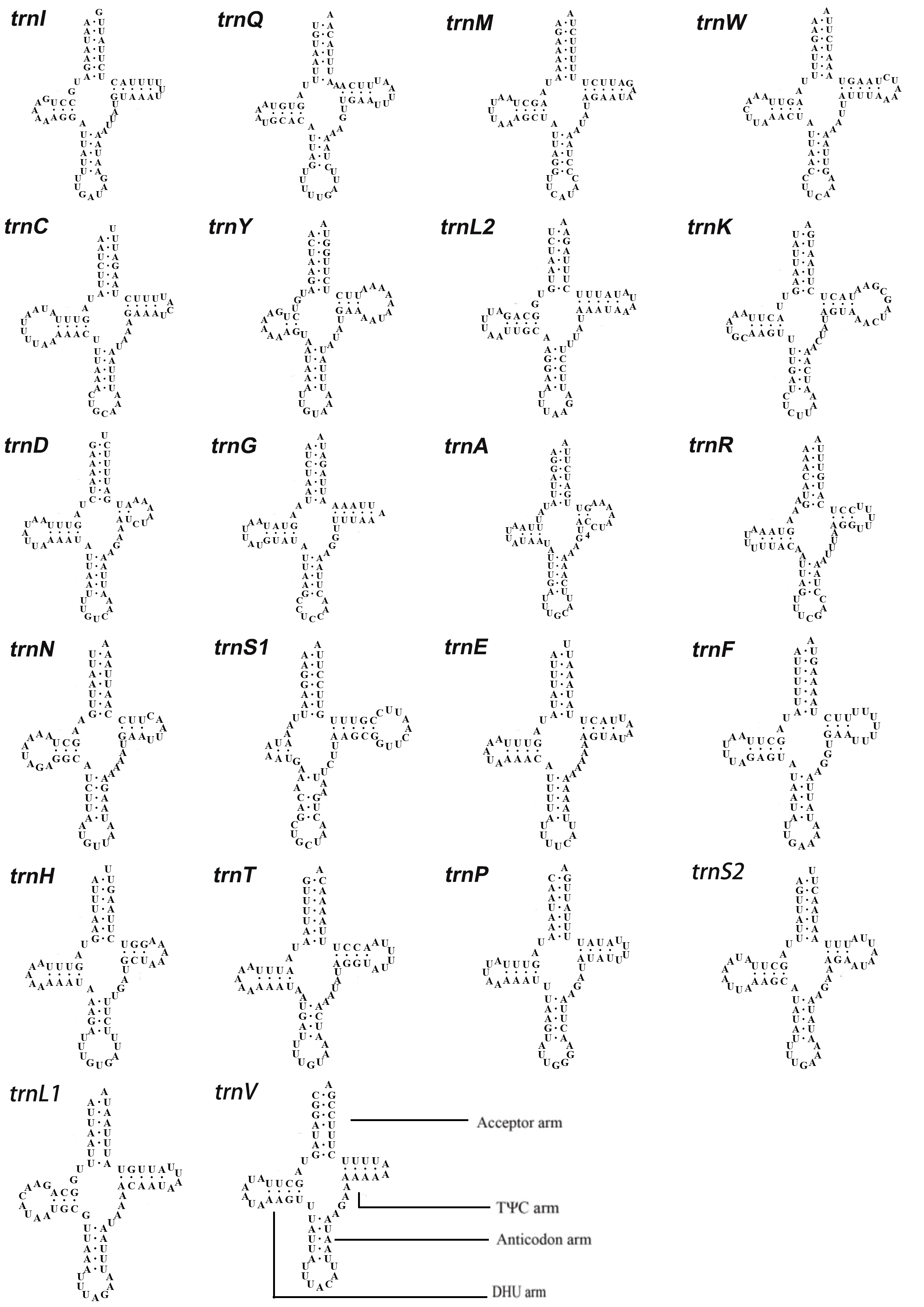

Supplement: Supplementary file 5 — Figure S5. Forecasted secondary structures of the 22 transfer RNAs (tRNAs) found in the mitogenome of Mileewa digitata dy. [file ECE3-15-e70830-s023.tif]

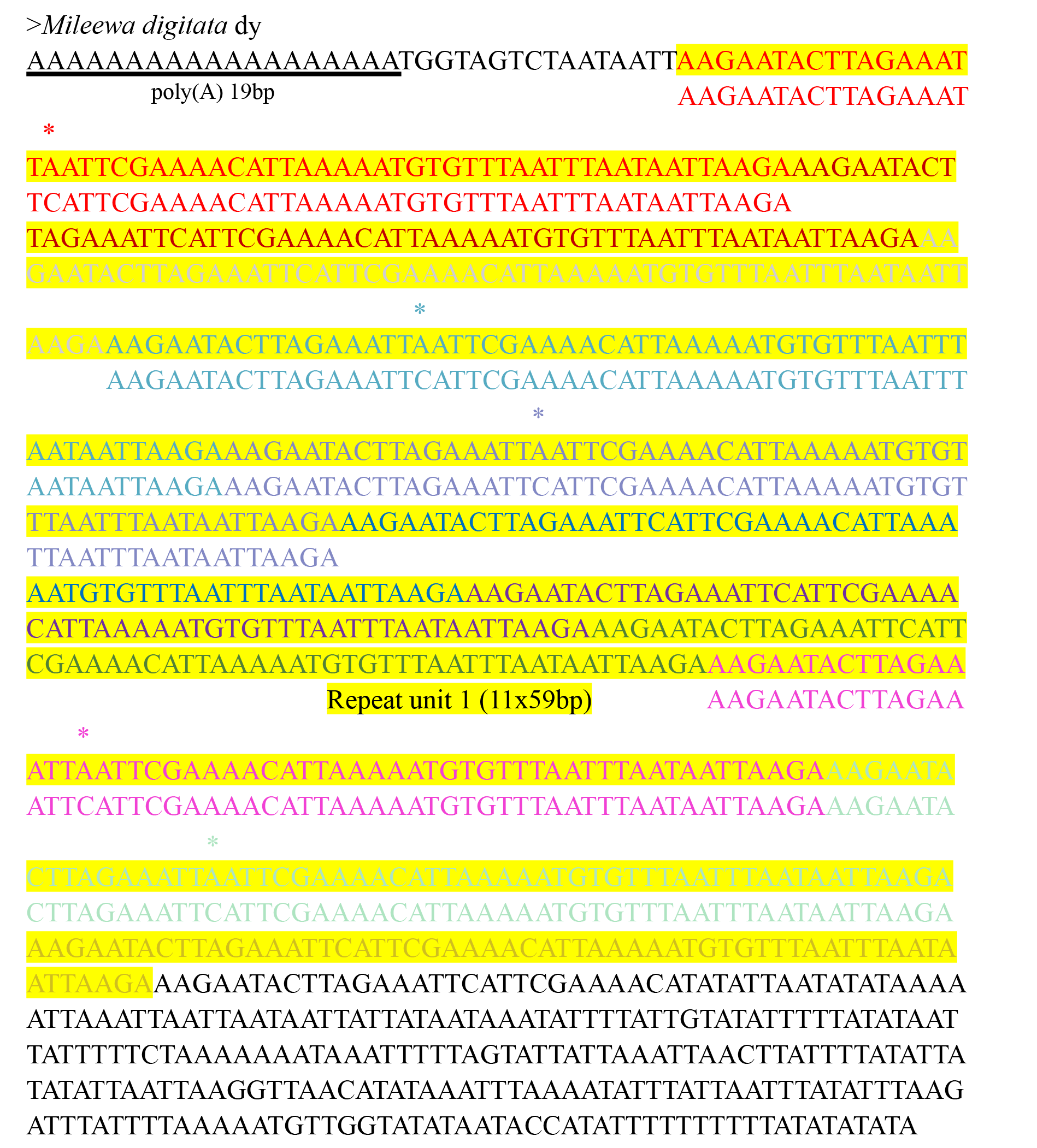

Supplement: Supplementary file 6 — Figure S6. Structure of the control region in the mitogenome of Mileewa digitata dy. Various repetitive sequences within the tandem repeat unit are denoted by distinct colors, while an asterisk (*) indicates a mismatch. [file ECE3-15-e70830-s027.tif]

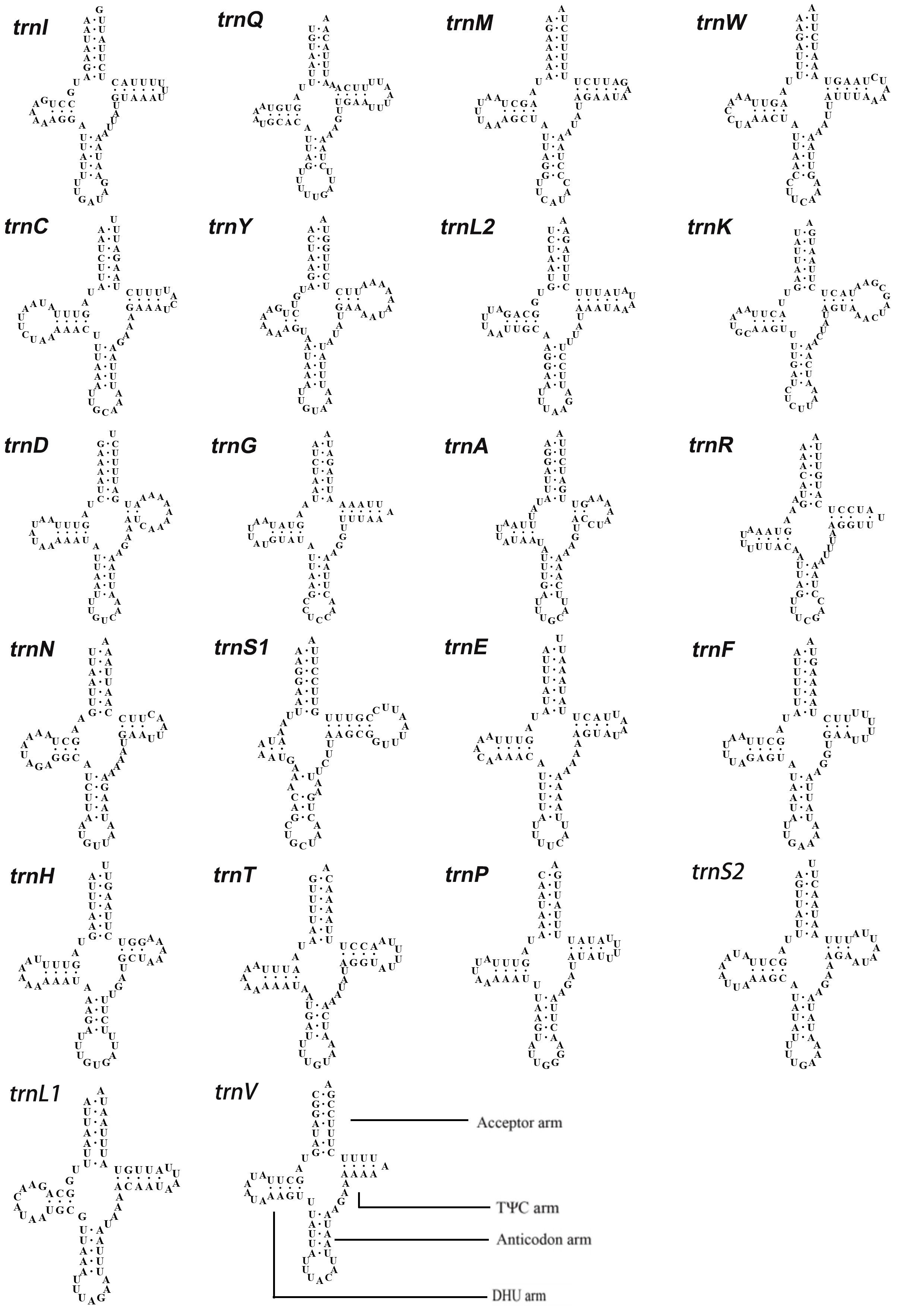

Supplement: Supplementary file 7 — Figure S7. Forecasted secondary structures of the 22 transfer RNAs (tRNAs) found in the mitogenome of Mileewa digitata hy. [file ECE3-15-e70830-s010.tif]

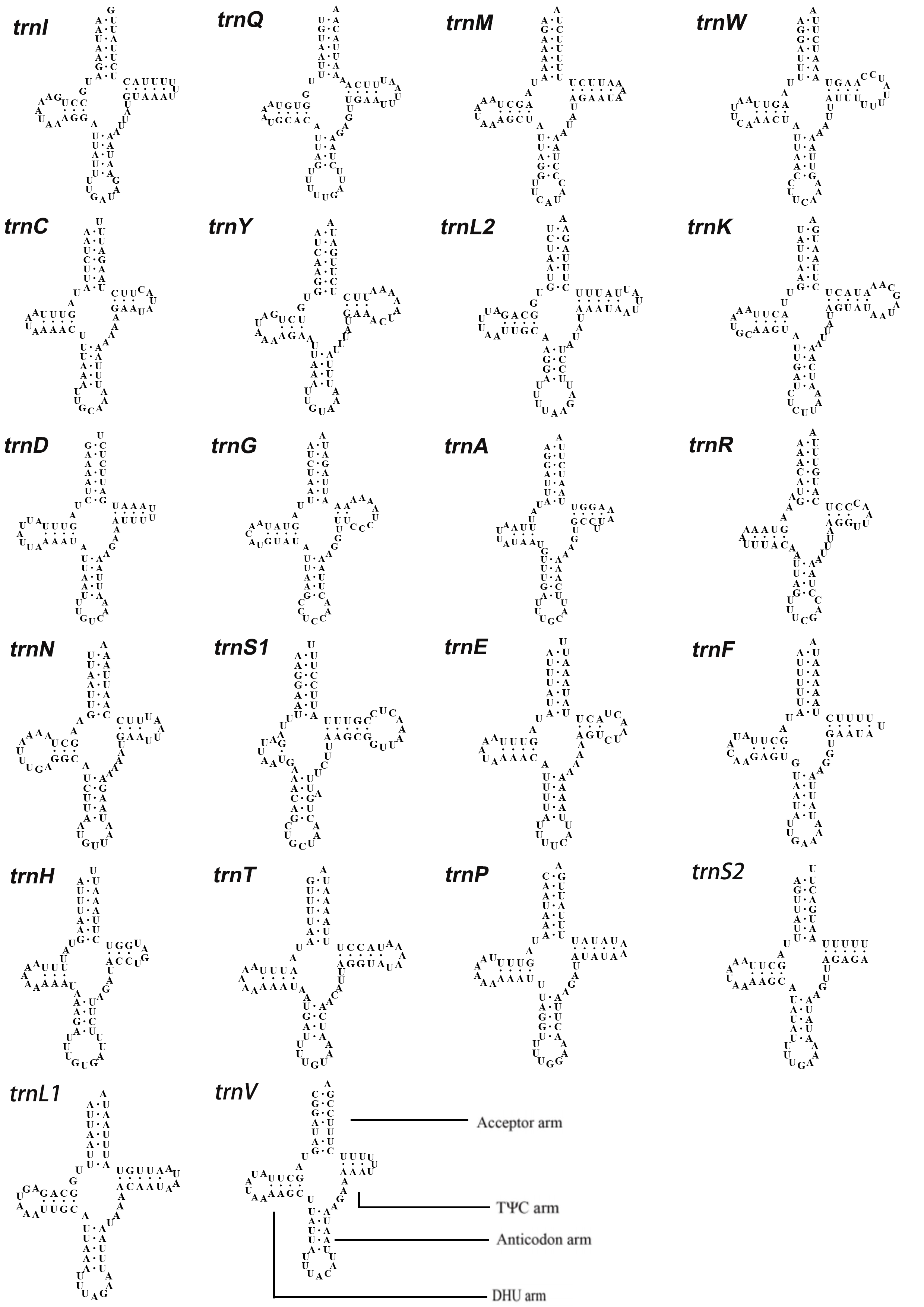

Supplement: Supplementary file 8 — Figure S8. Forecasted secondary structures of the 22 transfer RNAs (tRNAs) found in the mitogenome of Mileewa anchora. [file ECE3-15-e70830-s019.tif]

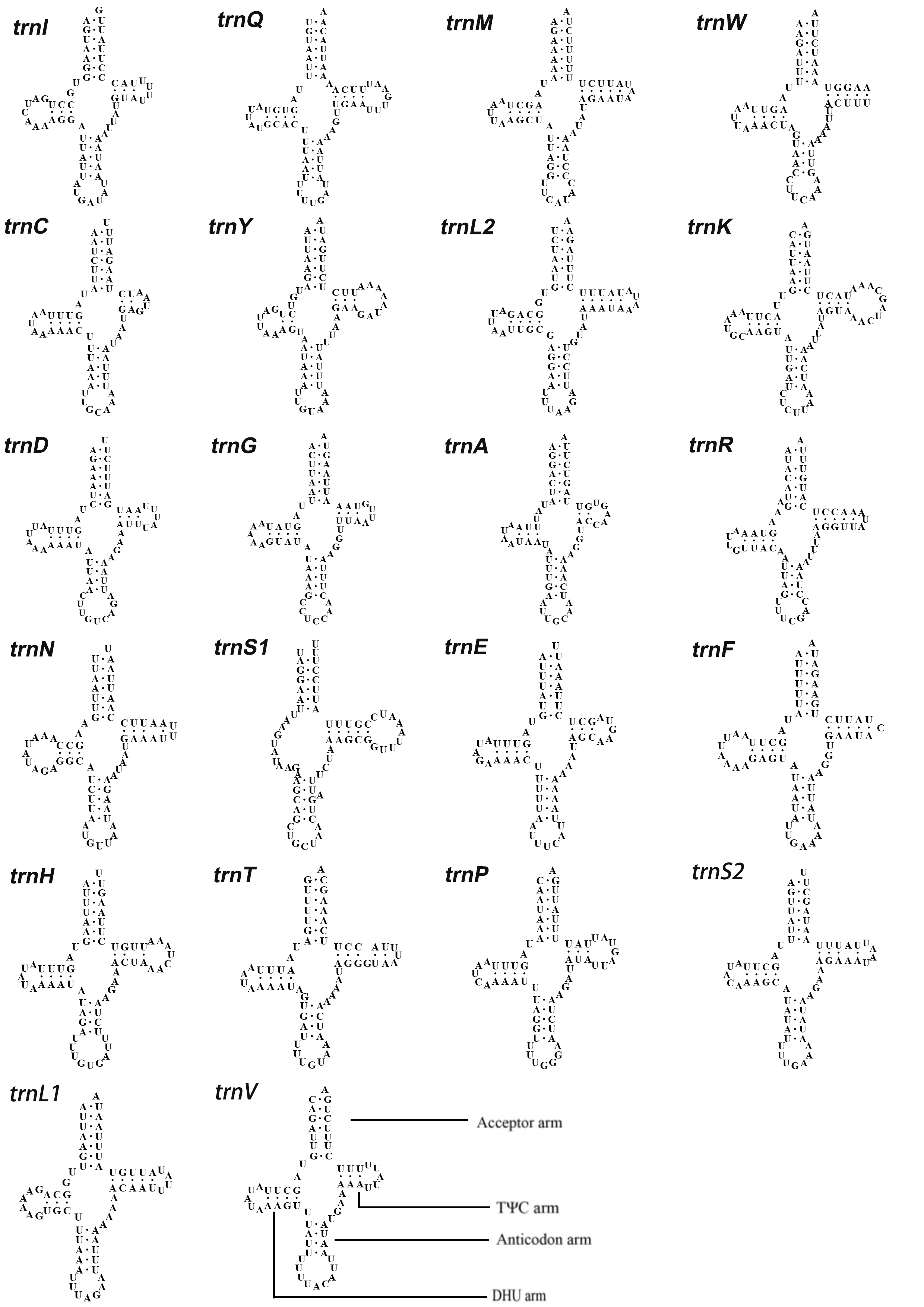

Supplement: Supplementary file 9 — Figure S9. Forecasted secondary structures of the 22 transfer RNAs (tRNAs) found in the mitogenome of Mileewa branchiuma. [file ECE3-15-e70830-s006.tif]

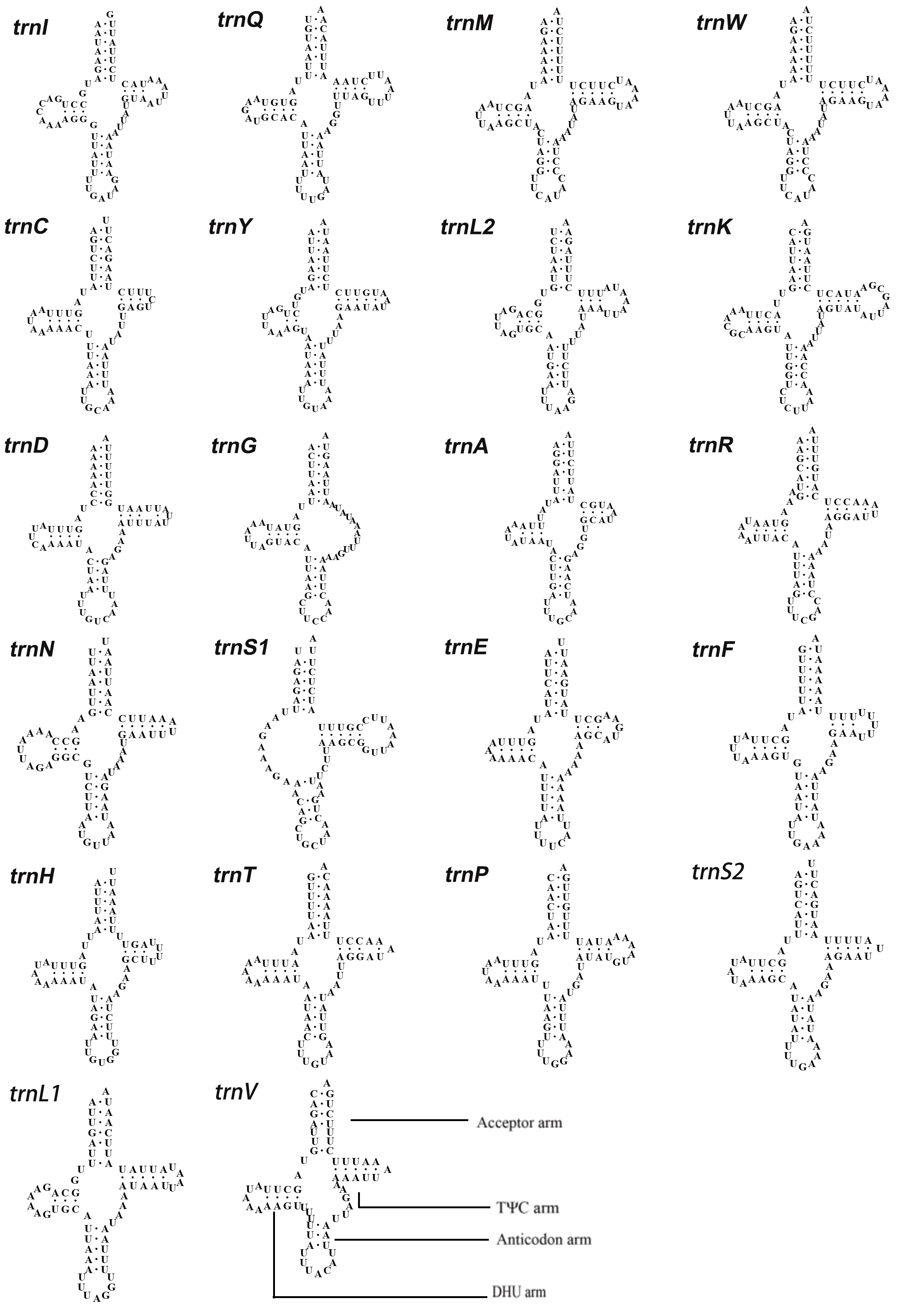

Supplement: Supplementary file 10 — Figure S10. Forecasted secondary structures of the 22 transfer RNAs (tRNAs) found in the mitogenome of Mileewa damingana. [file ECE3-15-e70830-s015.tif]

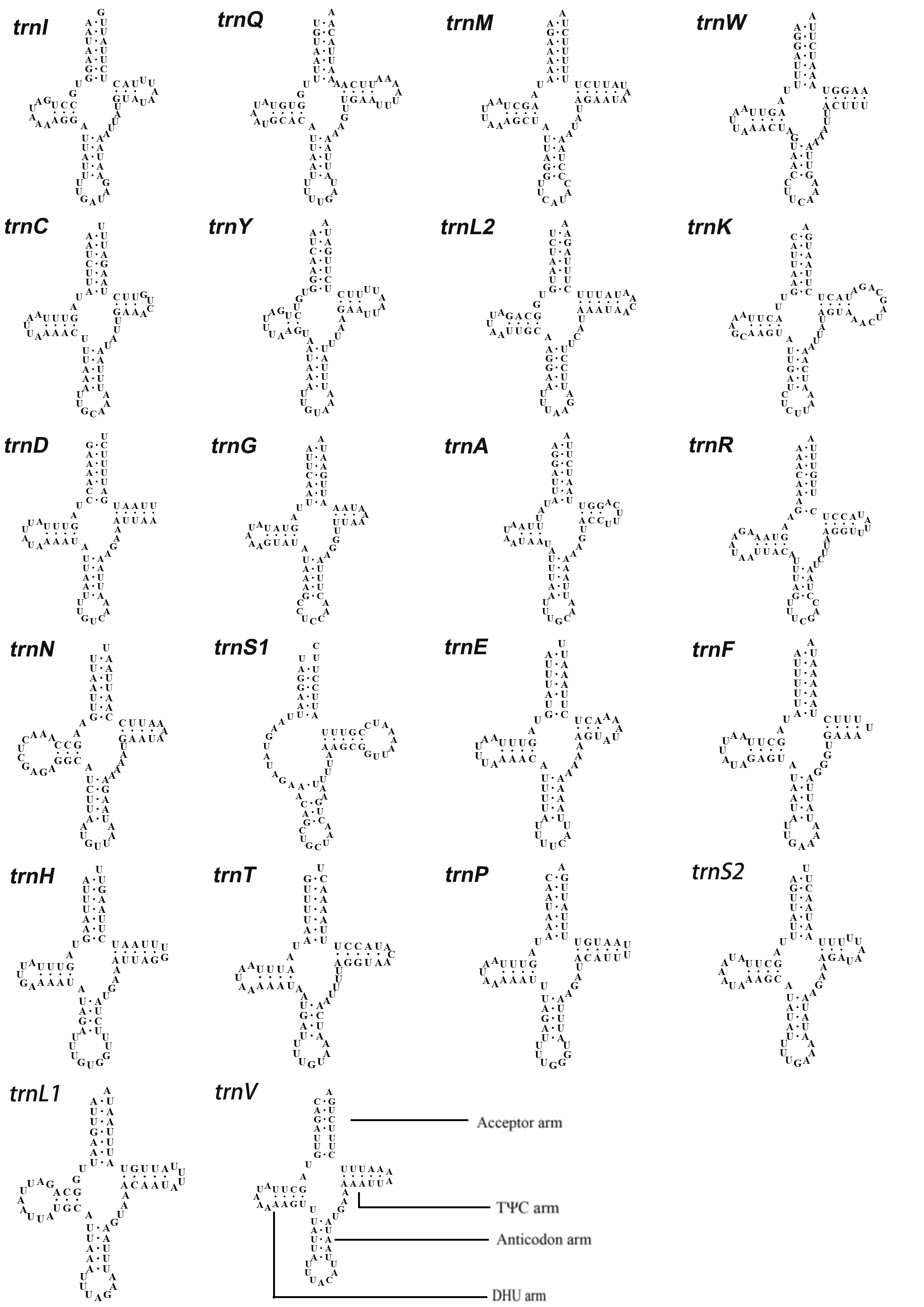

Supplement: Supplementary file 11 — Figure S11. Forecasted secondary structures of the 22 transfer RNAs (tRNAs) found in the mitogenome of Mileewa exsertocaputa. [file ECE3-15-e70830-s026.tif]

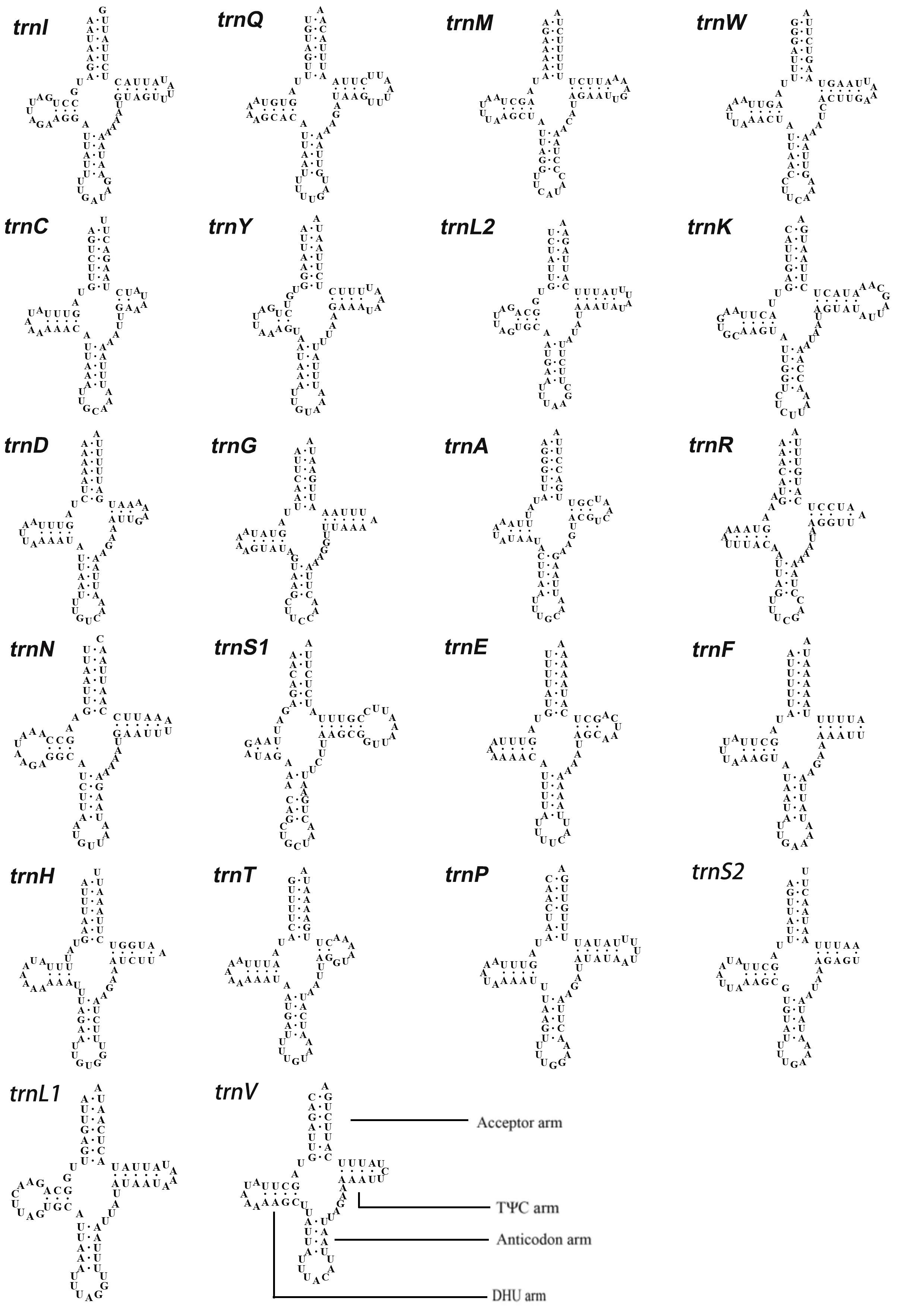

Supplement: Supplementary file 12 — Figure S12. Forecasted secondary structures of the 22 transfer RNAs (tRNAs) found in the mitogenome of Mileewa gaoligongana. [file ECE3-15-e70830-s020.tif]

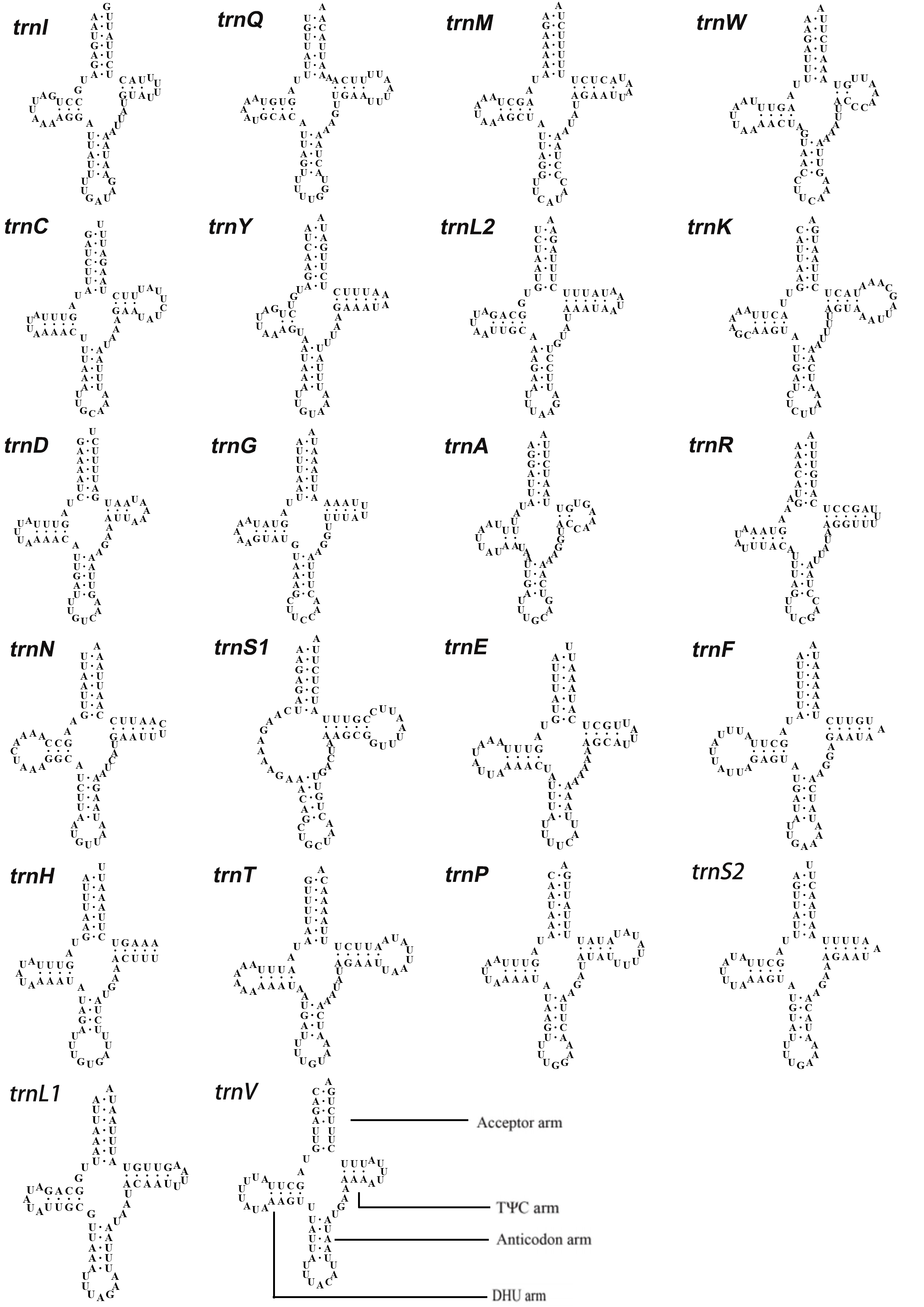

Supplement: Supplementary file 13 — Figure S13. Forecasted secondary structures of the 22 transfer RNAs (tRNAs) found in the mitogenome of Mileewa holomacula. [file ECE3-15-e70830-s007.tif]

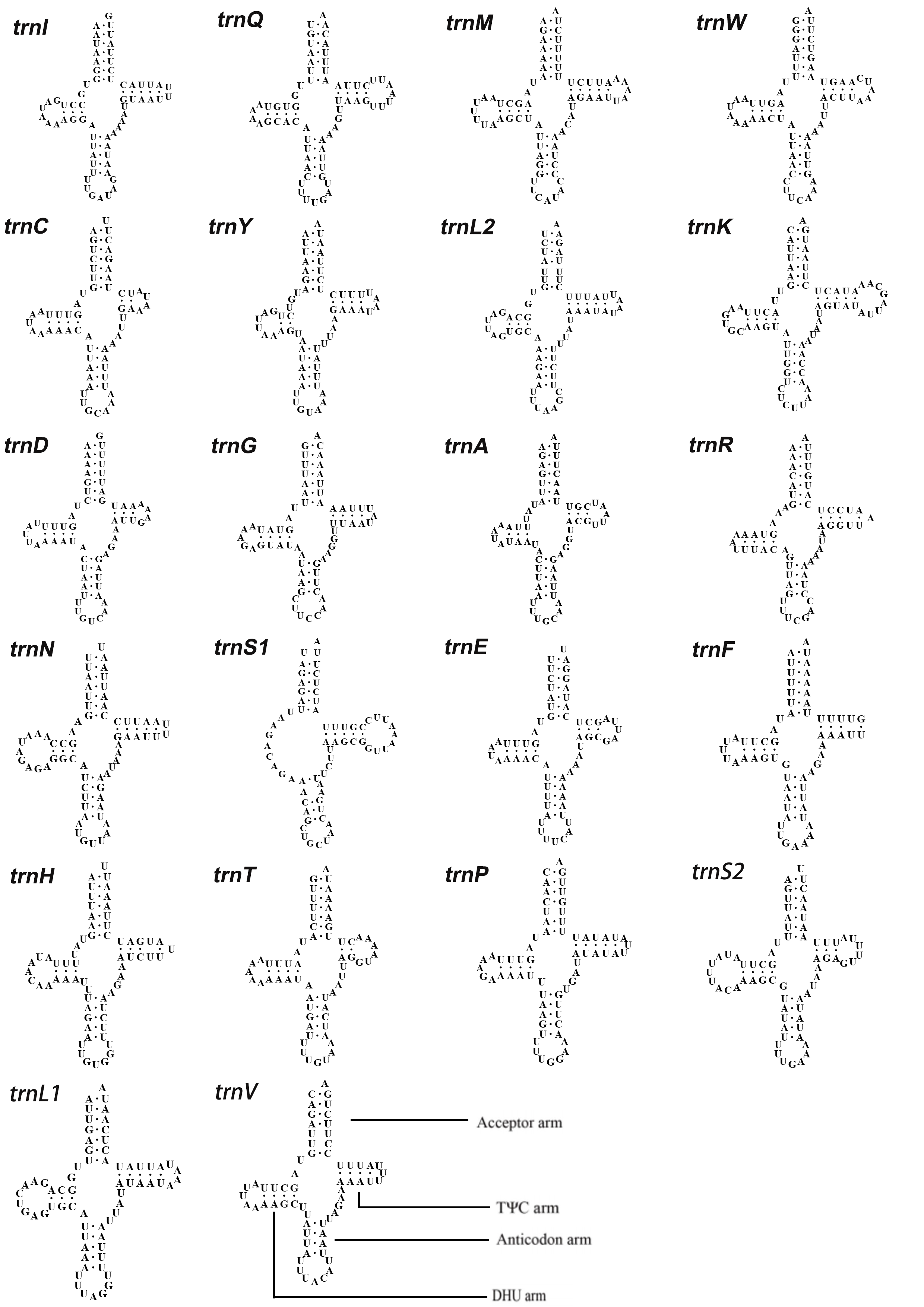

Supplement: Supplementary file 14 — Figure S14. Forecasted secondary structures of the 22 transfer RNAs (tRNAs) found in the mitogenome of Mileewa jinpingana. [file ECE3-15-e70830-s004.tif]

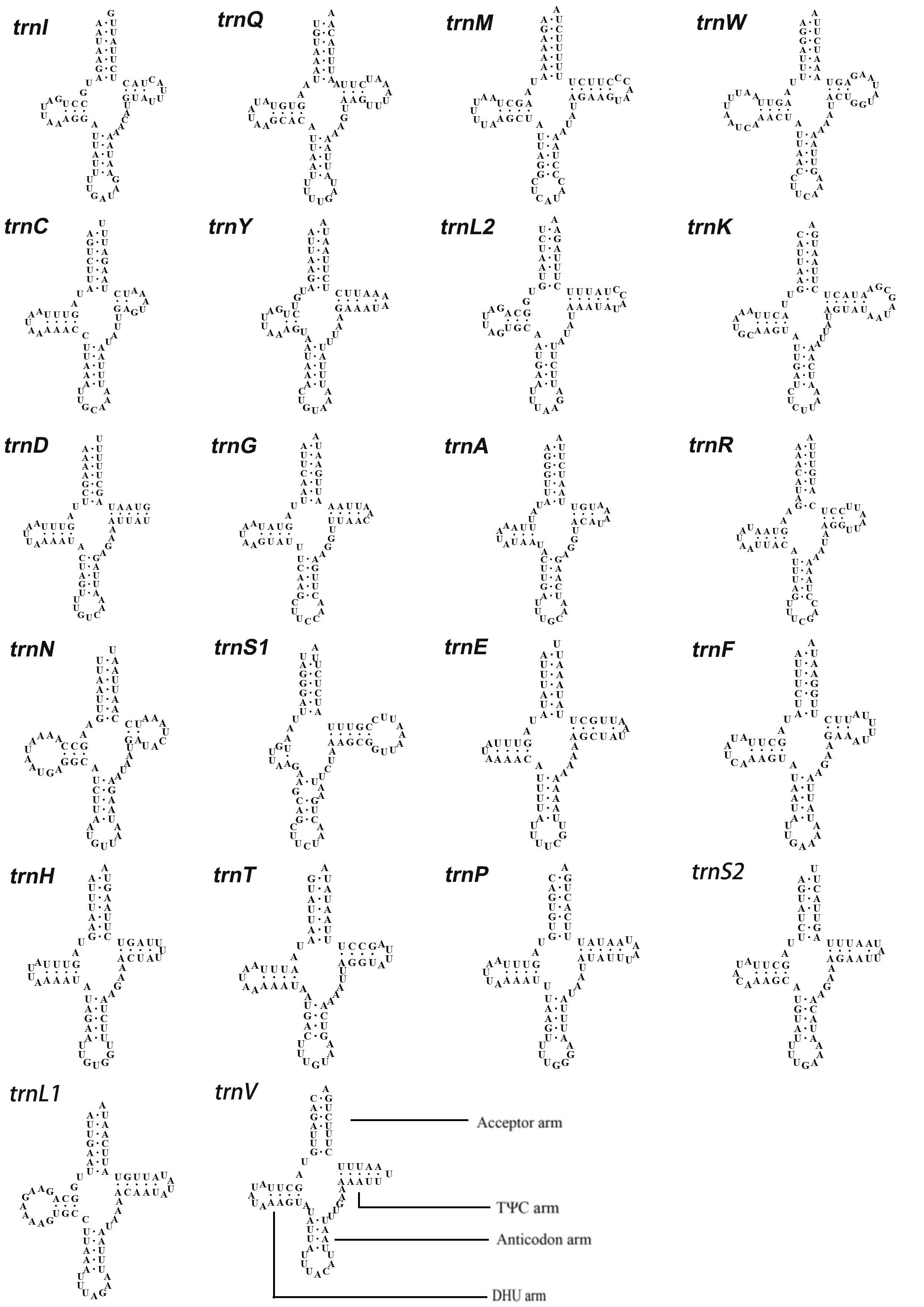

Supplement: Supplementary file 15 — Figure S15. Forecasted secondary structures of the 22 transfer RNAs (tRNAs) found in the mitogenome of Mileewa lackstripa. [file ECE3-15-e70830-s017.tif]

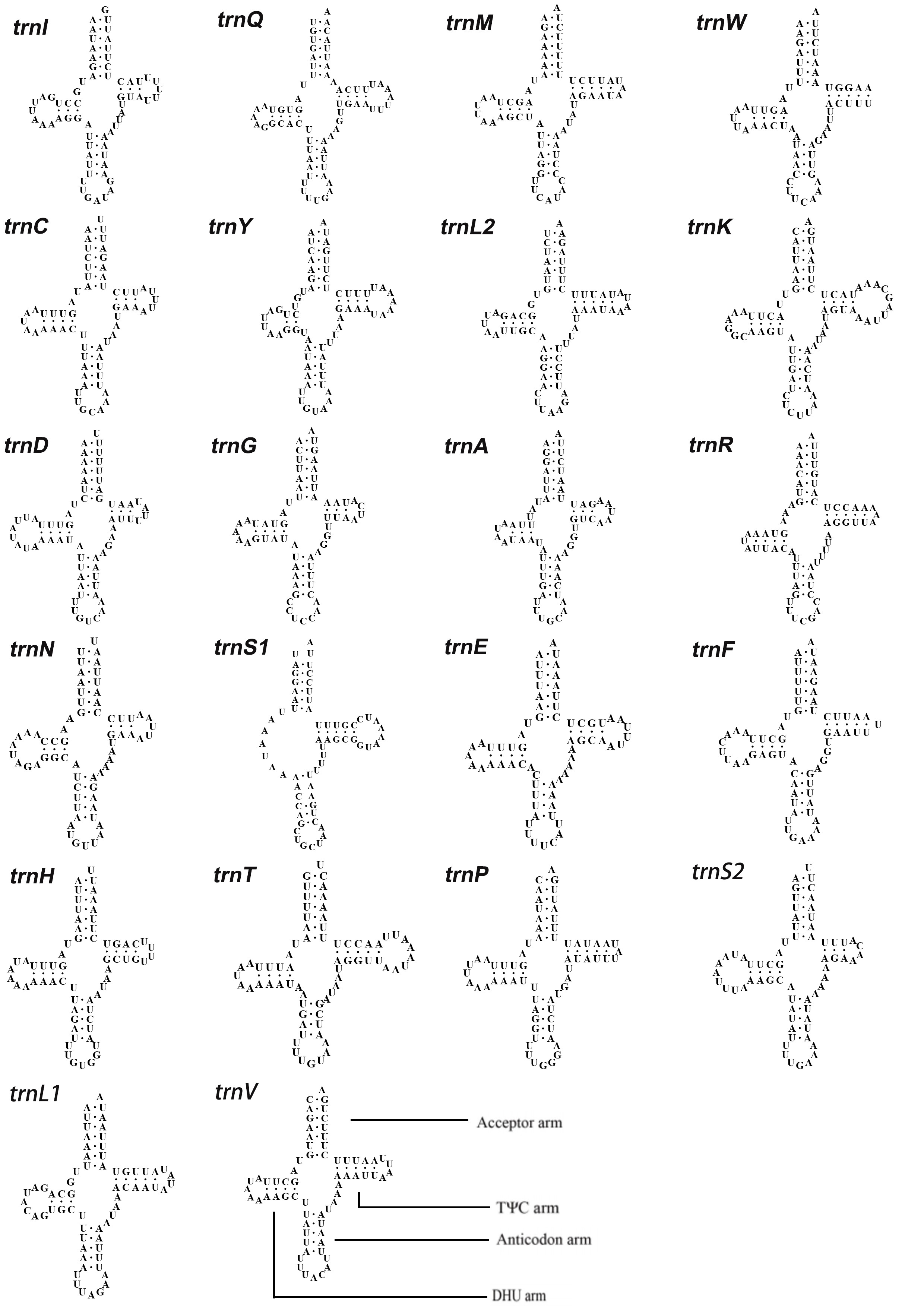

Supplement: Supplementary file 16 — Figure S16. Forecasted secondary structures of the 22 transfer RNAs (tRNAs) found in the mitogenome of Mileewa lynchi. [file ECE3-15-e70830-s025.tif]

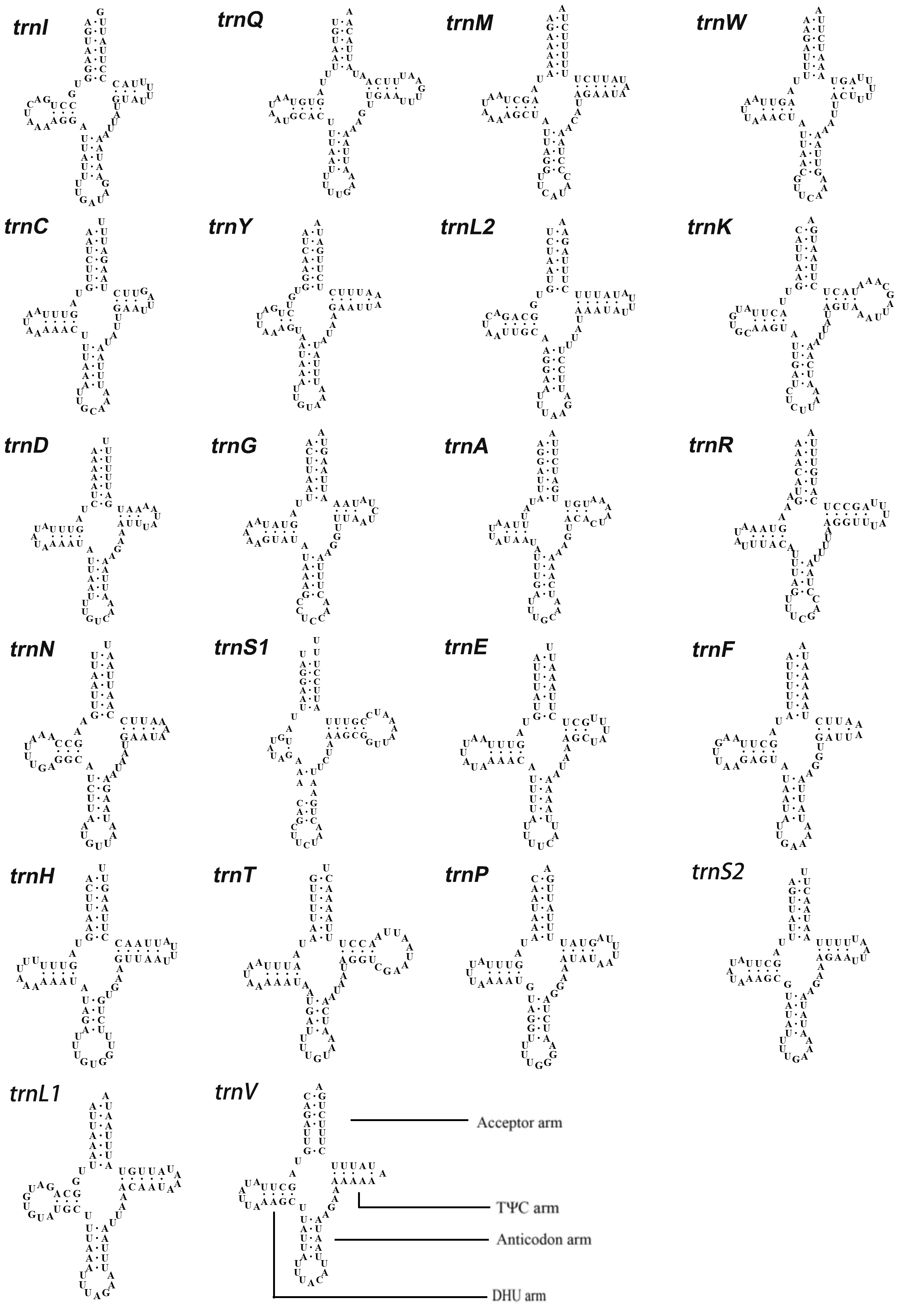

Supplement: Supplementary file 17 — Figure S17. Forecasted secondary structures of the 22 transfer RNAs (tRNAs) found in the mitogenome of Mileewa nii. [file ECE3-15-e70830-s012.tif]

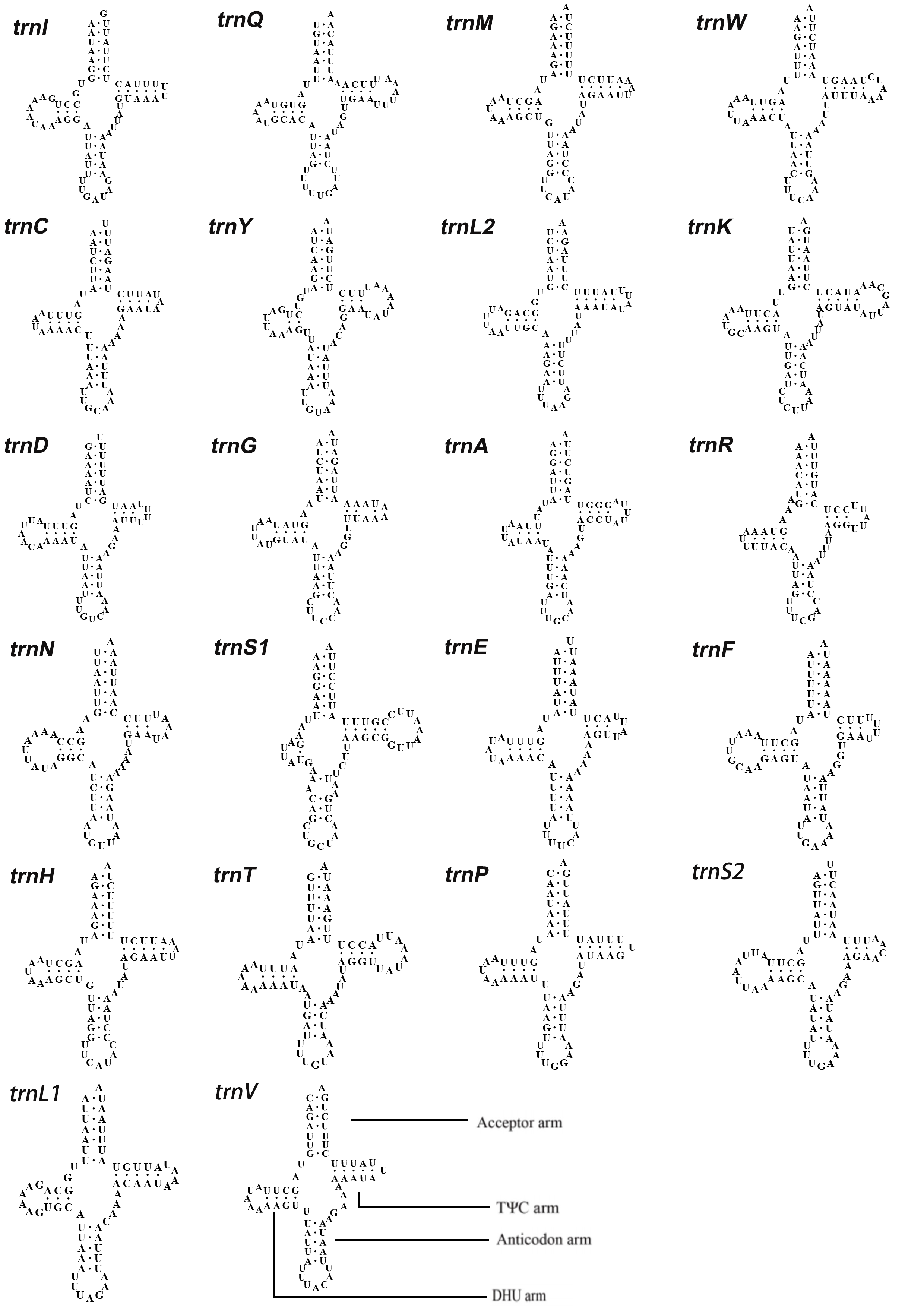

Supplement: Supplementary file 18 — Figure S18. Forecasted secondary structures of the 22 transfer RNAs (tRNAs) found in the mitogenome of Mileewa polymorpha. [file ECE3-15-e70830-s016.tif]

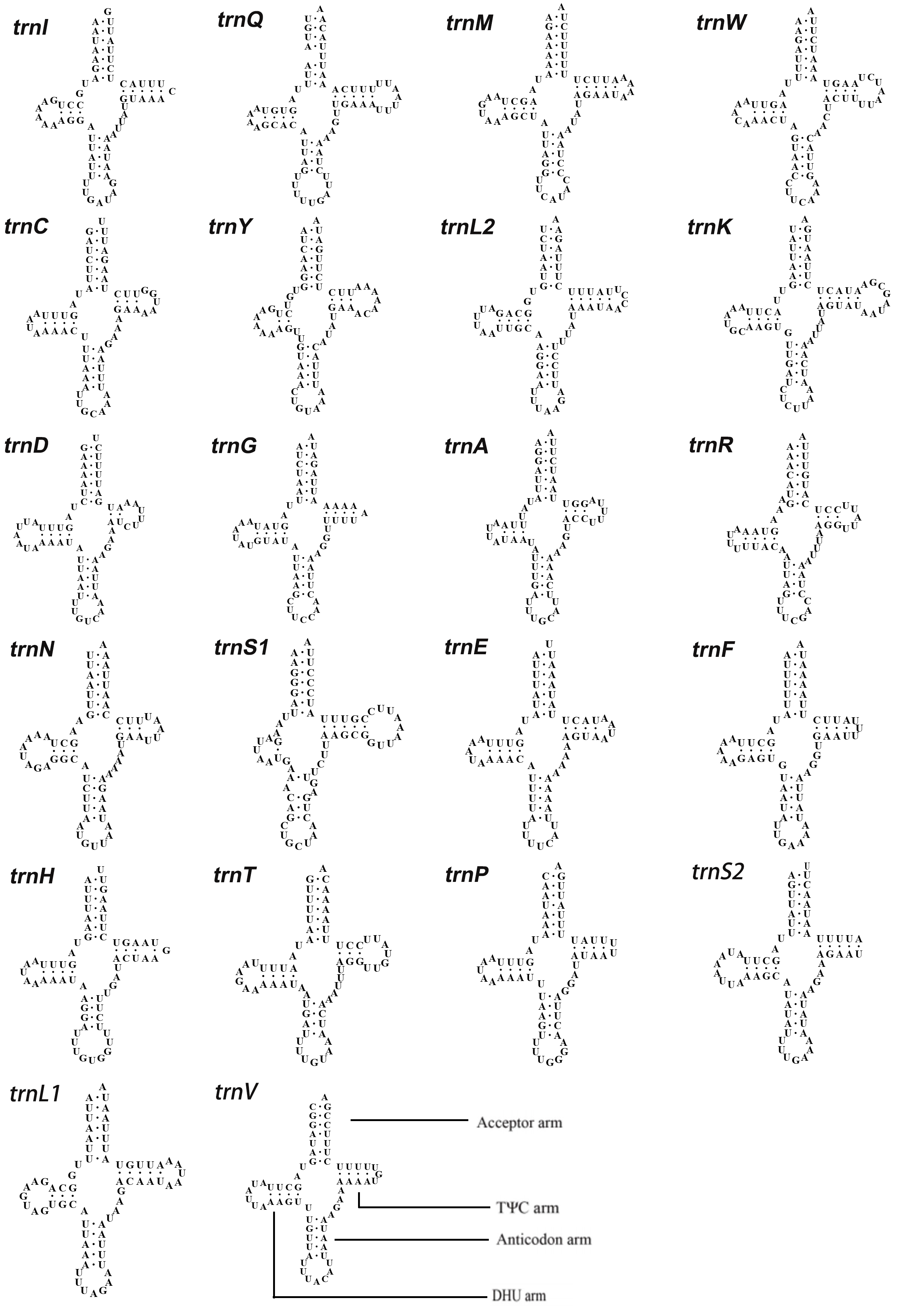

Supplement: Supplementary file 19 — Figure S19. Forecasted secondary structures of the 22 transfer RNAs (tRNAs) found in the mitogenome of Mileewa decemspina. [file ECE3-15-e70830-s002.tif]

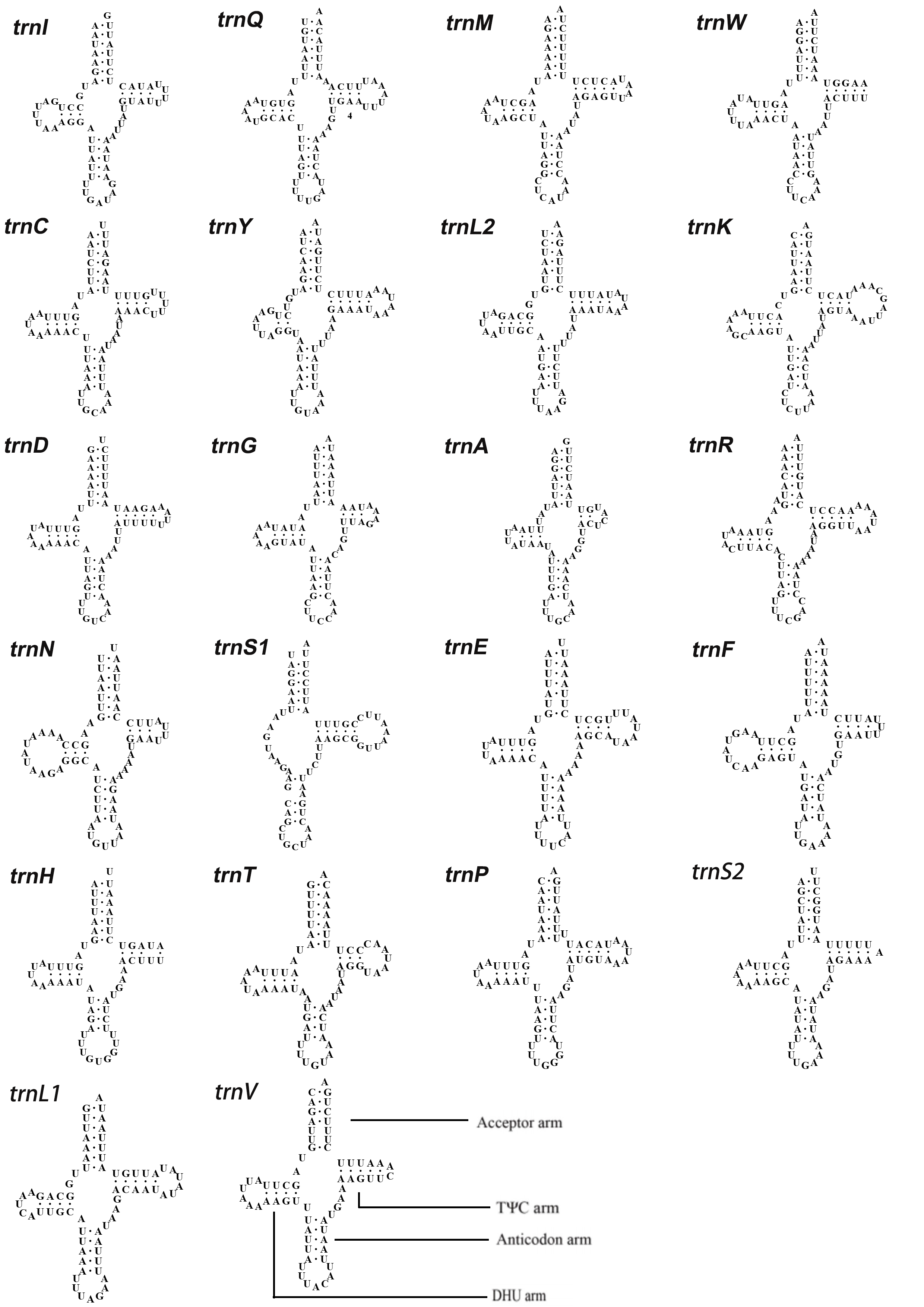

Supplement: Supplementary file 20 — Figure S20. Forecasted secondary structures of the 22 transfer RNAs (tRNAs) found in the mitogenome of Mileewa ussurica. [file ECE3-15-e70830-s024.tif]

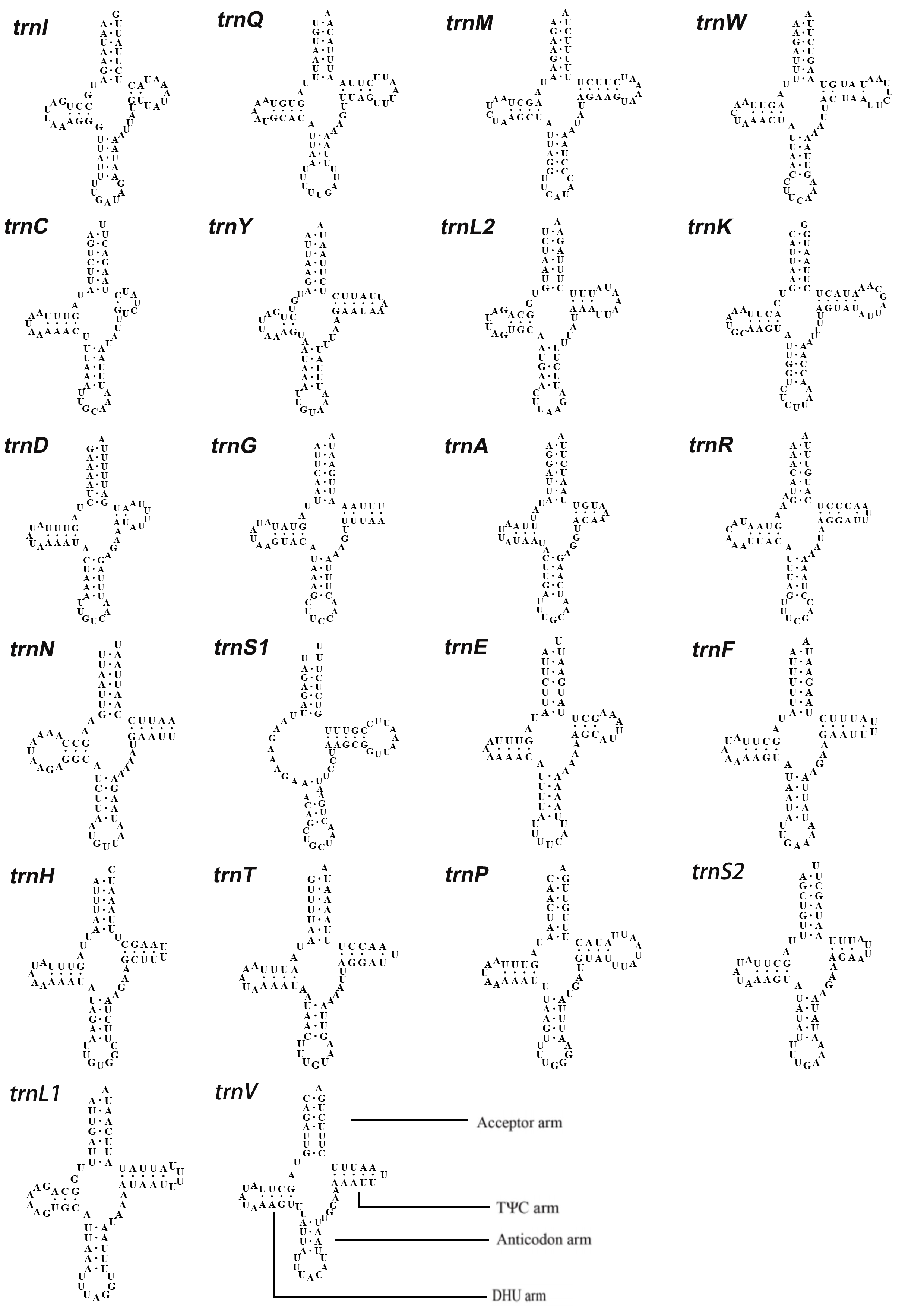

Supplement: Supplementary file 21 — Figure S21. Forecasted secondary structures of the 22 transfer RNAs (tRNAs) found in the mitogenome of Mileewa zhangi. [file ECE3-15-e70830-s011.tif]

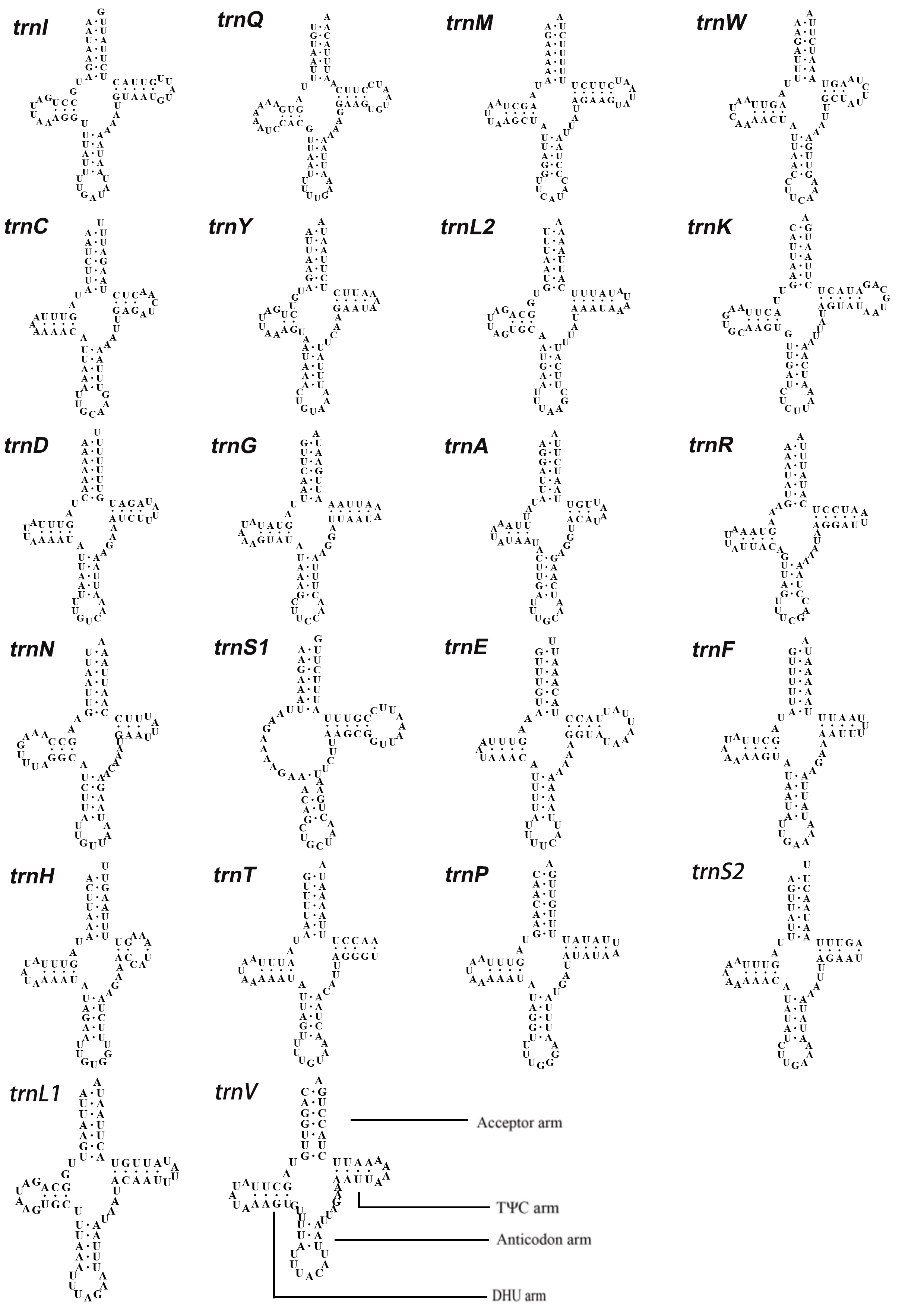

Supplement: Supplementary file 22 — Figure S22. Forecasted secondary structures of the 22 transfer RNAs (tRNAs) found in the mitogenome of Ujna liangae. [file ECE3-15-e70830-s003.tif]

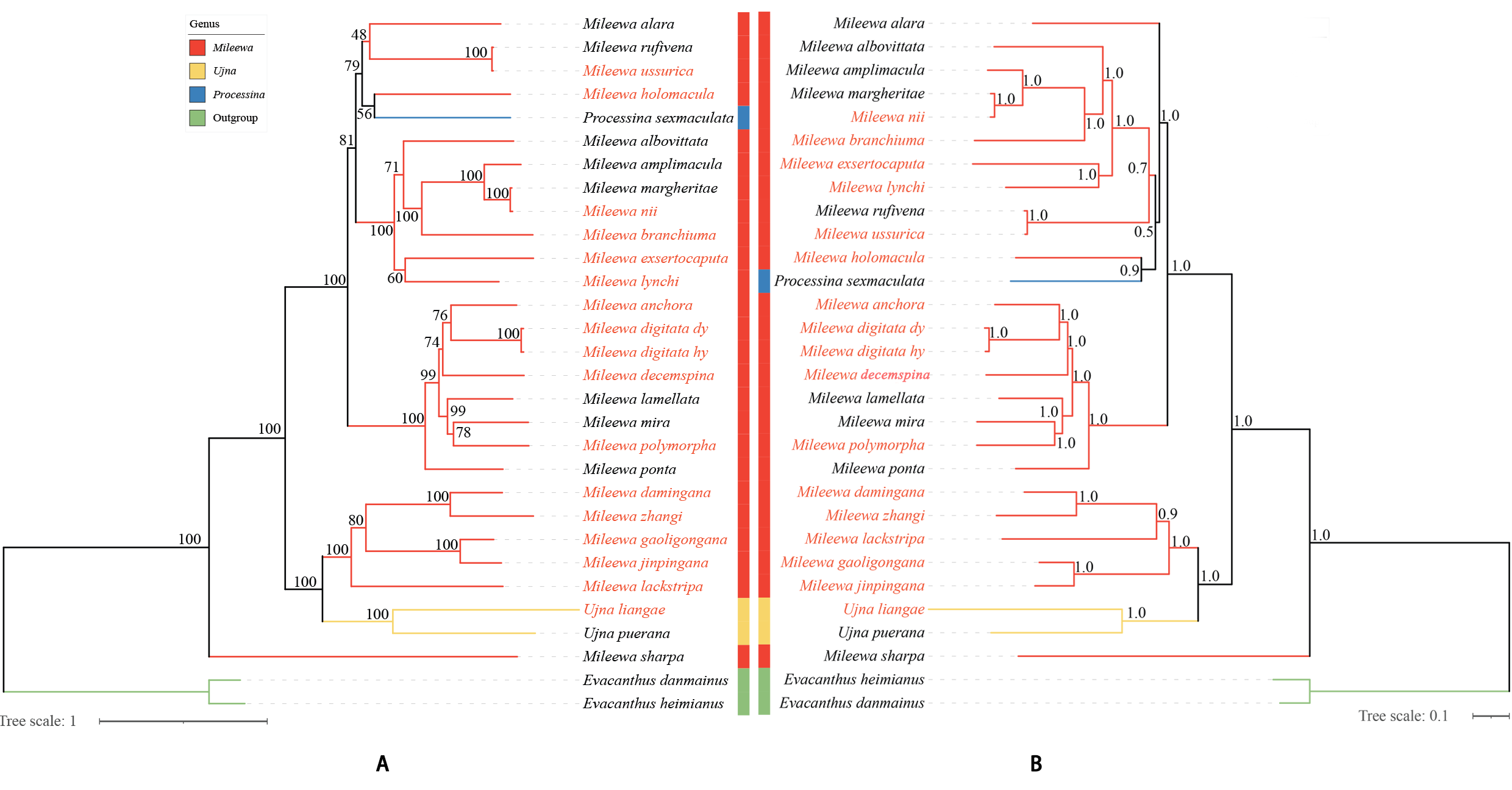

Supplement: Supplementary file 23 — Figure S23. Phylogenetic tree inferred from the PCGsRNA data set using Maximum Likelihood (ML) and Bayesian Inference (BI) methods. Bootstrap support (BS) and posterior probability (PP) values are displayed at the nodes of the trees. Mitogenomes that were newly sequenced are highlighted in red. (A) Maximum likelihood (ML) tree constructed from the PCGsRNA data set. (B) Bayesian inference (BI) tree generated from the PCGsRNA data set. PCGsRNA: 13 protein‐coding genes (PCGs) and 2 ribosomal RNA genes (rRNAs). [file ECE3-15-e70830-s018.tif]

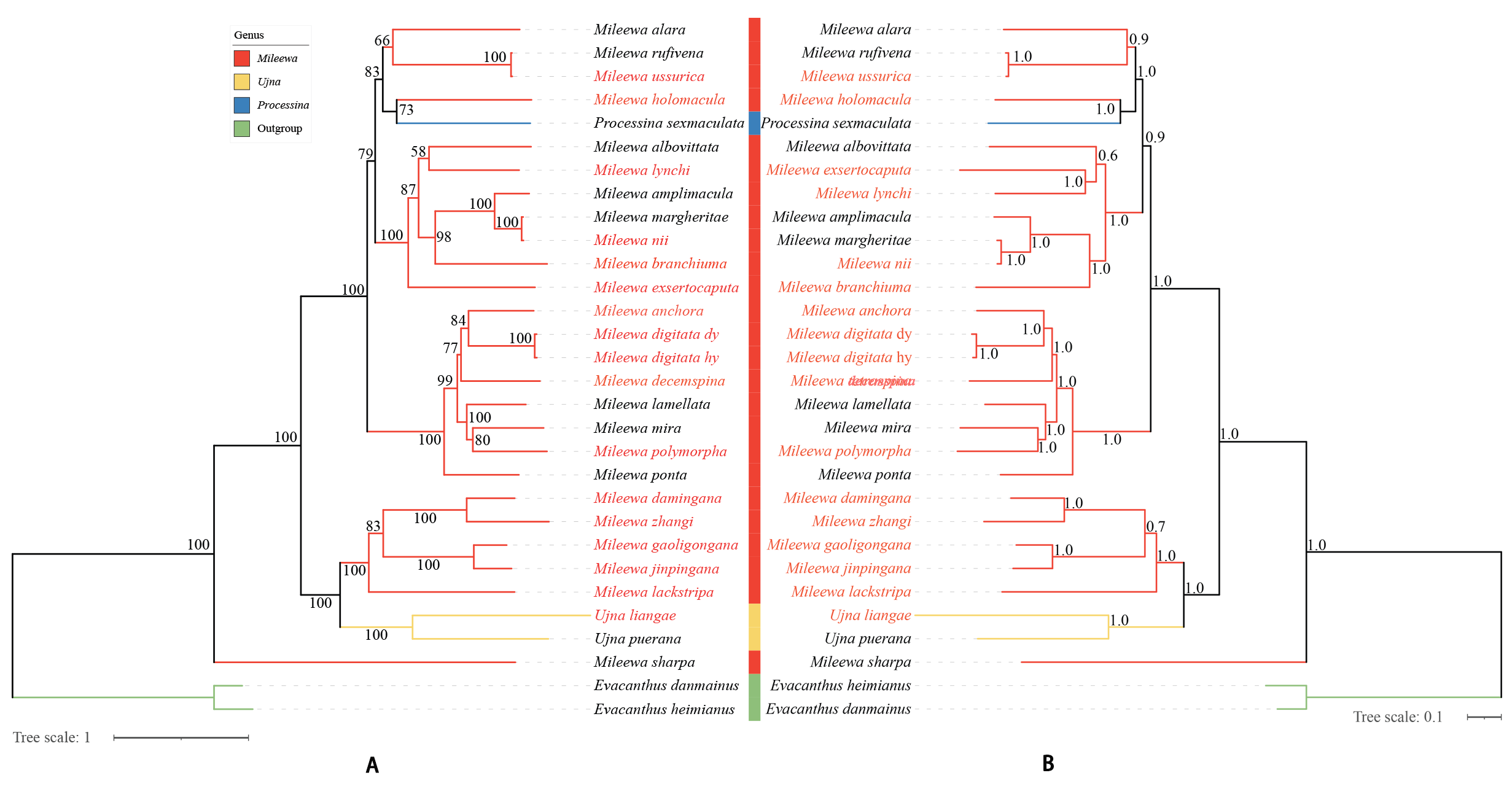

Supplement: Supplementary file 24 — Figure S24. Phylogenetic tree inferred from the PCGs data set using Maximum Likelihood (ML) and Bayesian Inference (BI) methods. Bootstrap support (BS) and posterior probability (PP) values are displayed at the nodes of the trees. Mitogenomes that were newly sequenced are highlighted in red. (A) Maximum Likelihood (ML) tree constructed from the PCGs data set. (B) Bayesian inference (BI) tree generated from the PCGs data set. PCGs: 13 protein‐coding genes (PCGs). [file ECE3-15-e70830-s021.tif]

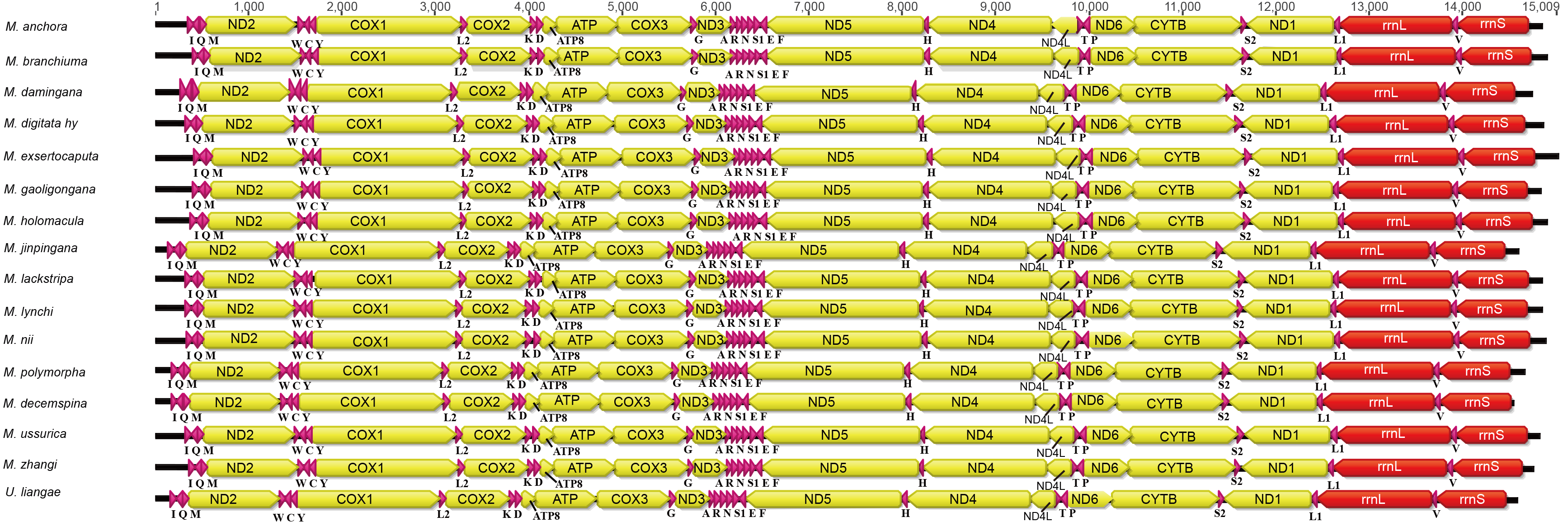

Supplement: Supplementary file 25 — Figure S25. Gene arrangement of 16 newly sequenced mitochondrial genomes of Mileewinae species. The rectangular blocks indicating the genes point right or left to denote their location on the heavy strand (H‐strand) and light strand (L‐strand), respectively. [file ECE3-15-e70830-s009.tif]

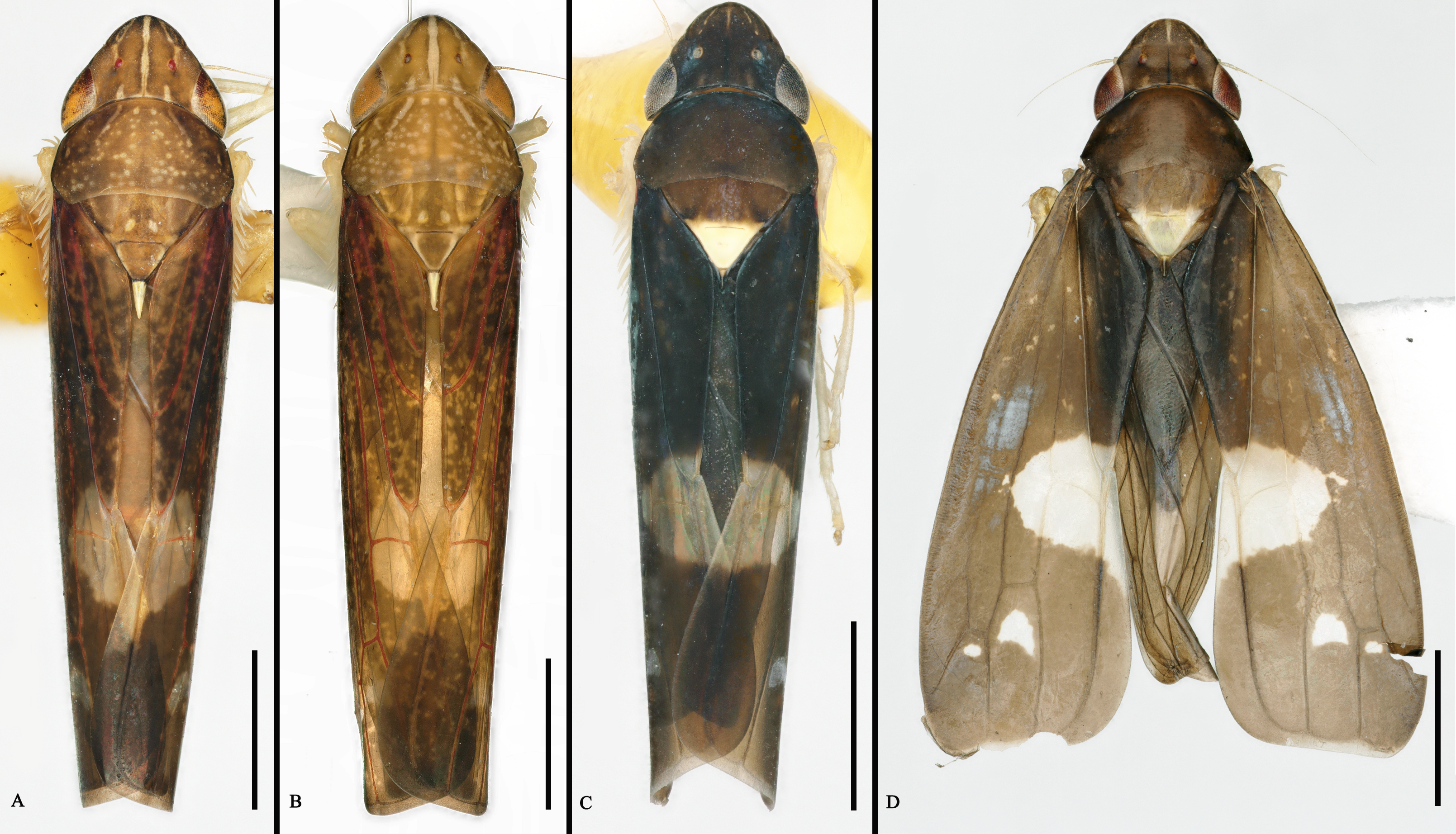

Supplement: Supplementary file 26 — Figure S26. Two pairs of morphologically similar species in this study. A, Mileewa rufivena. B, Mileewa ussurica. C, Mileewa Margheritae. D, Mileewa nii. [file ECE3-15-e70830-s014.tif]
